# Understanding the Bifunctional Trends of Fe‐Based Binary Single‐Atom Catalysts

**DOI:** 10.1002/advs.202301566

**Published:** 2023-06-21

**Authors:** Ruisong Li, Peng Rao, Daoxiong Wu, Jing Li, Peilin Deng, Zhengpei Miao, Xinlong Tian

**Affiliations:** ^1^ State Key Laboratory of Marine Resource Utilization in South China Sea Hainan Provincial Key Lab of Fine Chemistry School of Chemical Engineering and Technology Hainan University Haikou 570228 China

**Keywords:** bifunctional activity, binary single‐atom catalysts, synergistic effects, volcano relationship, zinc–air battery

## Abstract

Binary single‐atom catalysts (BSACs) have demonstrated fascinating activities compared to single atom catalysts (SACs) for oxygen reduction reaction (ORR) and oxygen evolution reaction (OER). Notably, Fe SACs is one of the most promising ORR electrocatalysts, and further revealing the synergistic effects between Fe and other 3d transition metals (M) for FeM BSACs are very important to enhance bifunctional performance. Herein, density functional theory (DFT) calculations are first adapted to demonstrate the role of various transition metals on the bifunctional activity of Fe sites, and a notable volcano relationship is established through the generally accepted adsorption free energy that Δ*G* ^*^_OH_ for ORR, and Δ*G* ^*^_O_−Δ*G* ^*^_OH_ for OER, respectively. Further, ten of the atomically dispersed FeM anchored on nitrogen‐carbon support (FeM‐NC) are successfully synthesized with typical atomic dispersion by a facile movable type printing method. The experimental data confirms the bifunctional activity diversity of FeM‐NC between the early‐ and late‐ transition metals, agrees very well with the DFT results. More importantly, the optimal FeCu‐NC shows the expected performance with high ORR and OER activity, thereby, the assembled rechargeable zinc–air battery delivers a high power density of 231 mW cm^−2^, and an impressive stability that can be stably operated over 300 h.

## Graphical abstract

A notable volcano relationship is established between the catalytic activity and the adsorption free energy for Fe‐based binary single‐atom catalysts with the addition of the early‐ or late‐transition metals. In addition, the performance trends of the bifunctional oxygen reduction reaction and oxygen evolution reaction are demonstrated by the experiments.

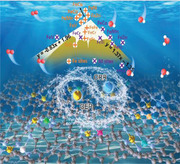

## 1 Introduction

Single atom catalysts (SACs) have been paid considerable attention as a hot frontier in heterogeneous catalysis owning to their huge atomic utilization efficiencies, remarkable electronic and atomic property.^[^ ^1^, ^2^, ^3^, ^4^, ^5^ ^]^ Among various SACs, the atomically dispersed Fe catalysts have demonstrated excellent activity toward oxygen reduction reaction (ORR),^[^ ^6^, ^7^, ^8^ ^]^ in which the geometrical Fe‐N_x_ structures are regarded as efficient active sites.^[^ ^9^ ^]^ Moreover, it has been reported that the Fe SACs can meet the requirements of non‐precious metal catalysts toward commercial fuel cells proposed by EIA at 2018.^[^ ^10^ ^]^ Despite its outstanding ORR properties, the electronic structure modulation of Fe‐based sites in the Fe SACs is still limited by inflexible Fe‐N_x_ configuration, leading to the difficulty in structure and activity engineering.^[^ ^11^, ^12^ ^]^ It is believed that the dilemma can be overcome through binary single‐atom catalysts (BSACs), which involves adjacent atom pairs and affords flexible modification depending on metallic compositions and synergistic effects, thus may further boost the activity.^[^ ^13^, ^14^, ^15^ ^]^ As presented for many BSACs, such as FeNi,^[^ ^16^ ^]^ CoNi,^[^ ^17^ ^]^ PtFe,^[^ ^18^ ^]^ CuZn,^[^ ^19^ ^]^ etc., have been synthesized and further verified better activity than the single SACs counterpart.

Take insight into the improving mechanism, remarkable charge density gradient and enlarged local torque can be found on heteronuclear BSACs, which delivers great capacity to promote catalytic performance and multifunctional capacity.^[^ ^20^, ^21^ ^]^ Additionally, the BSACs can efficiently increase the metal loadings, but also break the traditional linear scaling for the transition state of the asymmetric binary active sites.^[^ ^22^, ^23^, ^24^ ^]^ However, the current efforts mainly focus on a limited kinds of Fe‐based BSACs toward the ORR or oxygen evolution reaction (OER),^[^ ^25^, ^26^ ^]^ which results in the difficulty to realize effective bifunctional regulation. For instance, Wan et al. reported that FeNi BSACs exhibited higher OER activity than that of the Fe SACs, while a lower ORR activity was endowed in the former.^[^ ^27^ ^]^ As presented, little work has been contributed to systemically reveal the corresponding relationship between bifunctional performance and various components, especially for the early transition metals. One big challenge is that the atomically dispersed atoms not only tend to aggregation because of thermodynamic instability, but also deliver different coordination capacity for different metal atoms, both of which leads to the impediment and invalidation in synthesizing a series of Fe‐based BSACs.^[^ ^28^, ^29^, ^30^ ^]^ In addition, the correlation between the bimetallic centers and the mechanistic pathways of the bifunctional reactions have remained unsolved, which limits the practical applications of Fe‐based BSACs. Therefore, developing a general strategy to synthesize various Fe‐based BSACs, and revealing the synergistic effects between Fe and another isolated atom toward bifunctional catalysis are significant to design more efficient catalysts.

Herein, the activity of FeM BSACs (M = early transition metals (Sc, Ti, V, Cr) and late transition metals (Mn, Fe, Co, Ni, Cu and Zn)) toward bifunctional ORR and OER were first investigated by density functional theory (DFT) calculations, thereby, a large difference for the synergistic effects on the Fe sites with a model of FeM‐N_6_ has been revealed, and a notable volcano relationship can be established with the early‐ and late‐transition metals. Meanwhile, ten representative FeM‐NC with high‐density isolated atoms were synthesized via a movable type printing method, which employed C_3_N_4_ with six long electron pairs to stabilize metal ions as metallic precursors, and polydopamine containing abound amino and hydroxyl groups can bind heteronuclear atomic pairs and further anchor metal atoms as support after pyrolysis. The electrochemical data demonstrated that the early transition metals can exhibit unsatisfactory effects on bifunctional activity of the FeM‐NC, while a positive tendency can be obtained for the late transition metals, which agrees very well with the DFT calculations. Remarkably, a low voltage gap of 0.63 V between ORR and OER, and superior long‐term stability can be achieved in the optimal FeCu‐NC catalyst, which also shows a power density of 231 mW cm^−2^ and high cycling stability at the zinc–air battery level.

## 2 Results and Discussions

### 2.1 Theoretical Activities of FeM BSACs

First, we conducted the DFT calculations to obtain the bifunctional ORR and OER activity of the FeM‐BSACs, and revealed the synergistic effects between Fe and M atoms on the activity of Fe sites. The atomic structure adopted in our calculations is displayed in Figure S1 (Supporting Information), where two transition metal atoms coordinating with six nitrogen atoms to form the FeM‐N_6_ moieties. Previous works have shown that such FeM‐N_6_C moieties are experimentally accessible.^[^ ^31^ ^]^ It is considered that the Sc≈Fe sites in FeM‐N_6_C are covered by an OH intermediate via a self‐adjusting mechanism when served as reaction sites.

**Figure 1:**
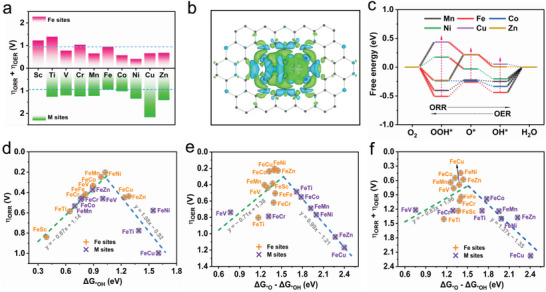
Theoretical investigations. a) Theoretical overpotential gap between OER and ORR (*η*_ORR_ + *η*_ORR_) of FeM‐BSACs. Note that the O intermediate is not stably adsorbed to the Sc site and tends to migrate to the vicinity of the Fe site, thus we cannot obtain the *η*_ORR_ and *η*_ORR_ of Sc sites. b) Difference charge density of FeNi‐N_6_C defined as *Δρ*=*ρ*_total_ − *ρ*_NC_ − *ρ*_Ni_ − *ρ*_Fe_, where *ρ*_total_, *ρ*_NC_, *ρ*_Ni_, and *ρ*_Fe_ are the charge density of FeNi‐N_6_C, NC support, Ni site, and Fe site, respectively. The iso‐surface value is adopted as 0.001 e bohr^−1^.^[^ ^3^ ^]^ Blue and green represent positive and negative iso‐surface, respectively. c) Calculated free energy diagram of Fe sites in FeMn‐, FeFe‐, FeCo‐, FeNi‐, FeCu‐ and FeZn‐N_6_C. The ORR and OER potential‐determining steps are indicated by translucent black and red line segments, respectively. d) Volcano relationship of the *η*_ORR_ versus ΔG_*OH_, e) *η*_OER_ versus ΔG_*O_ − ΔG_*OH_, and f) *η*_ORR_ + *η*_ORR_ versus ΔG_*O_ − ΔG_*OH_.

Free energies of the four‐electron steps are calculated to evaluate the theoretical overpotential of ORR (*η*_ORR_) and OER (*η*_OER_). The catalysts adsorped with one OOH, O, and OH molecule are denoted as OOH^*^, O^*^, and OH^*^, respectively. The potential gap defined as *η*_ORR_ + *η*_OER_ is used to access the bifunctional activity of FeM‐N_6_C. As summarized in **Figure** **1**a and Table S1 (Supporting Information), the bifunctional activity of FeM‐N_6_C can be effectively modulated by choosing the M atoms. As displayed in Figure 1b, there is notably redistribution of charge density between the two transition metal sites, further reflecting the synergistic effects in the FeM‐N_6_C moieties. The major contributors to bifunctional oxygen reduction and evolution reactions are Fe sites in FeM‐N_6_C, except for the FeTi‐N_6_C, where the potential gap of Ti sites is smaller than that of Fe sites. Compared to FeFe‐N_6_C with *η*_ORR_ + *η*_OER_ of 0.95 V, FeV‐, FeMn‐, FeCo‐, FeNi‐, FeCu‐ and FeZn‐N_6_C exhibit higher bifunctional activity with smaller *η*_ORR_ + *η*_OER_ of 0.76, 0.65, 0.57, 0.41, 0.66, and 0.68 V, respectively. The free energy diagrams of Fe sites in FeM‐N_6_C are displayed in Figure 1c and Figure S2 (Supporting Information) The atomic structures of adsorption conformations are summarized in Figure S1 Supporting Information. The ORR and OER processes mediated by FeFe‐, FeV‐, FeMn‐, and FeCo‐N_6_C are limited by the desorption of OH^*^ and the formation of O_2_ molecule, respectively. Interestingly, the introduction of Ni, Cu, and Zn next to the Fe atoms leads to a significant increasement in the free energies of OOH^*^, O^*^, and OH^*^, thus changing the ORR and OER potential‐determining steps of the Fe sites. The ORR potential‐determining steps of FeNi‐, FeCu‐ and FeZn‐N_6_C are estimated to be the desorption of OH intermediates, the formation of OOH^*^, and formation of O^*^, respectively. For OER, the activity of FeNi‐N_6_C and FeCu‐N_6_C is limited by the formation of OOH^*^, while FeZn is limited by the formation of O^*^. Therefore, it can be concluded that M atoms of FeM‐BSACs plays an important role in enhancing or decreasing the activity of Fe sites.

According to the well‐known Sabatier principle,^[^ ^32^ ^]^ high catalytic activity requires the adsorption strength of intermediates on the active sites is neither too strong nor too weak. The Sabatier principle in the bifunctional FeM‐N_6_C can be expressed as several volcanic relationships related to the theoretical overpotential of ORR, OER, and bifunctional reactions depicted in Figure 1d–f, and the optimal catalytic activity is achieved at the volcano peak. The left and right hand of the volcano peak corresponds to strong and weak adsorption of intermediates, respectivley. Previous works show that the ΔG_*OH_ and ΔG_*O_ − ΔG_*OH_ are suitable catalytic descriptors for *η*_ORR_ and *η*_OER_, respectively.^[^ ^33^, ^34^ ^]^ Based on the fitting linear relationships, the optimal ΔG_*OH_ and ΔG_*O_ − ΔG_*OH_ are estimated as 1.06 and 1.52 eV for ORR and OER activity, respectively. Notably, it is found that the ΔG_*O_ − ΔG_*OH_ can serve as a descriptor for the bifunctional activity of FeM‐N_6_C, and the optimal ΔG_*O_ − ΔG_*OH_ value is calculated as 1.50 eV. The volcano's negative slope, representing large ΔG_*O_ − ΔG_*OH_, is made up of M sites that are relatively far from the volcano peak, corresponding to their low bifunctional activity. Compared to FeFe‐N_6_C, the combination of Fe and M (M = V, Mn, Co, Ni, Cu, and Zn) weakens the adsorption of O and OH intermediates on the Fe sites (Table S1, Supporting Information), which not only leads to a larger ΔG_*OH_ (≈0.89–1.28 eV) and enhanced ORR activity (Figure 1d), but also resulting in a suitable ΔG_*O_ − ΔG_*OH_ value (≈1.26–1.45 eV) with higher OER and bifunctional activity (Figure 1e,f). Based on the above discussions, two conclusions can be draw that: 1) the major active sites in the FeM‐N_6_C are Fe sites, and 2) enhanced bifunctional activity can be achieved on FeV‐, FeMn‐, FeCo‐, FeNi‐, FeCu‐, and FeZn‐N_6_C due to the synergistic effects between Fe and M atoms by modulating the adsorption free energy of the reatcion intermediates.

### 2.2 Synthesis and Characterization of FeM‐NC

**Figure 2:**
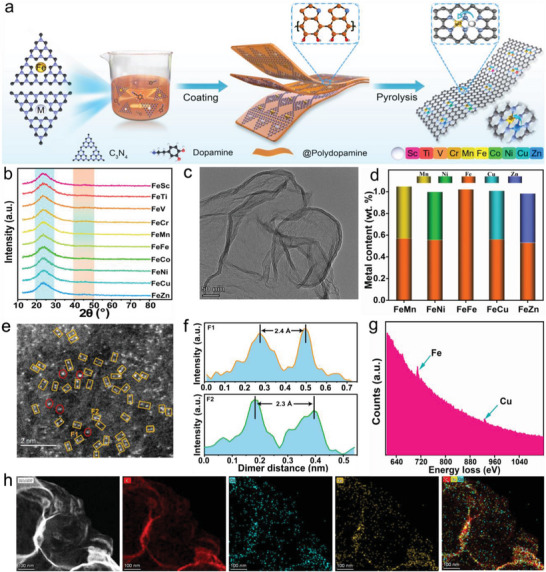
Synthesis and characterization of FeM‐NC. a) Schematic illustration of synthesizing FeM BSACs supported on NC (FeM‐NC), corresponding b) XRD patterns and c) TEM images. d) Metal contents of the FeMn‐NC,FeNi‐NC,FeFe‐NC,FeCu‐NC, and FeZn‐NC. e) AC‐STEM image (circle: very few single atoms; square: numerous binary atoms), f) intensity profile of binary atoms, g) EELS analysis of binary atoms for Fe and Cu elements and h) corresponding Fe and Cu mapping analysis for FeCu‐NC.

Since the atomically dispersed metals can be stabilized by their coordination with heteroatom species,^[^ ^35^, ^36^ ^]^ we employ the bulk C_3_N_4_ substances as stabilizer in the precursors, and nitrogen‐doping carbon (NC) as the support (**Figure** **2**a). In detail, various transition metals with the non‐granular state were evenly distributed on C_3_N_4_ substances (labeled as M‐C_3_N_4_, Figure S3, Supporting Information) as metallic precursors through the strong coordination capacity of the triazine ring in C_3_N_4_. Subsequently, these non‐granular metals with heteronuclear atom pairs were adsorbed by polydopamine, and further transferred on NC support derived from monolayer polydopamine after pyrolysis, thus generating the atomically dispersed FeM‐NC. It can be seen that no specific peaks (≈40–50°) of the 3d transition metals appear in X‐ray diffraction patterns (XRD), except for a similar nitrogen–carbon peak at ≈24°, suggesting no metallic nanoparticles in the ten representative FeM‐NC (Figure 2b). This result was further demonstrated by high‐magnification transmission electron microscopy (TEM) images, in which no obvious metallic nanoparticles can be observed (Figure 2c; S4 and S5, Supporting Information). Accordingly, these metals were highly dispersed with atomic state on the ultrathin NC support (Figure S6, Supporting Information), which possesses a thickness of approximately 2.6 nm (Figure S7, Supporting Information). Based on the positive activity effect of the late‐transition metals on FeM BSACs predicted by DFT analysis, five representative FeM‐NC (M = Mn, Ni, Fe, Cu and Zn) were employed as subjects to further analyze their composition and structure features by this strategy. A similar content of the 3d transition metals can be found in the five FeM‐NC by inductively coupled plasma mass spectrometry analysis (Figure 2d), which is consist with the X‐ray photoelectron spectroscopy results corresponding to an approximate atomic ratio of 1:1 for Fe and M (Table S2, Supporting Information). In addition, ≈12 at.% of nitrogen content suggests the high coordination capacity with metal atoms for the NC supports (Table S2, Supporting Information), which also are endowed with high graphitization degrees (Figure S8, Supporting Information). For structural features, microporous and mesoporous distributions are predominately fabricated with a high specific surface area at a range from 373–412 m^2^ g^−1^ in the five as‐obtained samples (Figure S9 and Table S2, Supporting Information). Remarkably, the aberration‐corrected scanning transmission electron microscopy (AC‐STEM) images clearly show the isolated Fe and M atoms in the five FeM‐NC (Figure 2e; Figure S10, Supporting Information). Especially for the targeted FeCu‐NC, few isolated Fe or M atoms (marked by the red circle) and numerous adjacent atom pairs (marked by the yellow square) can be observed (Figure 2e), and the latter presents a predominant dimer distance from 0.226 to 0.261 nm, allowing to generate the electron interaction between the adjacent atom pair (Figure 2f; Figure S11, Supporting Information). The atom pairs were further analyzed by the electron energy loss spectra (EELS), and two distinct peaks are identified as the Fe and Cu species, respectively (Figure 2g; Figure S12, Supporting Information), indicating the adjacent Fe‐Cu pairs in FeCu‐NC. In addition, the corresponding Fe and Cu mapping images suggest that the isolated atoms are dispersed on the surface of the NC support with different intensity distribution, but no overlapping and aggregating Fe and Cu atoms, implying that the dual‐metal atoms were anchored with heteronuclear pairs on monolayer NC (Figure 2h).

### 2.3 Spectroscopic Characterizations

X‐ray photoelectron spectroscopy (XPS), X‐ray absorption near edge structure (XANES), and extended X‐ray absorption fine spectra (EXAFS) were employed to identify the electronic state and local coordination chemistry of M and Fe atoms in FeM‐NC. The high‐resolution C 1s spectra of FeMn‐NC, FeNi‐NC, FeFe‐NC, FeCu‐NC and FeZn‐NC were similar to that of the NC support (Figure S13, Supporting Information). Interestingly, compared to the NC sample, the N 1s spectra of the five FeM‐NC samples presented an additional peak at 399.4 eV, indicating the existence of metal–nitrogen coordination (Figure S14, Supporting Information).^[^ ^37^ ^]^ For metallic coordination, two main peaks at 641.0 eV (2p _3/2_) and 652.8 eV (2p _1/2_) in Mn 2p spectrum (Figure S15a, Supporting Information), two main peaks at 855.4 eV (2p _3/2_) and 872.0 eV (2p _1/2_) in Ni 2p spectrum (Figure S15b, Supporting Information), four main peaks corresponding to Cu^+^ and Cu^2+^ in Cu 2p spectrum (Figure S15c, Supporting Information), and two main peaks at 1021.4 eV (2p _3/2_) and 1044.6 eV (2p _1/2_) in Zn 2p spectrum (Figure S15d, Supporting Information), suggesting the oxidation states of the 3d metals in FeM‐NC. The Fe 2p spectra also showed two characteristic peaks corresponding to Fe 2p _1/2_ and Fe 2p _3/2_ along with two satellite peaks (Figure S16, Supporting Information). However, after forming the adjacent Fe‐M pairs, the Fe 2p _3/2_ spectra of FeMn‐NC exhibited a slightly negative shift compared to that of Fe in FeFe‐NC, while the Fe 2p _3/2_ spectra of FeNi‐NC, FeCu‐NC and FeZn‐NC display an increasing positive shift, suggesting that the electronic interaction between Fe and M atoms of the FeM‐NC.

**Figure 3:**
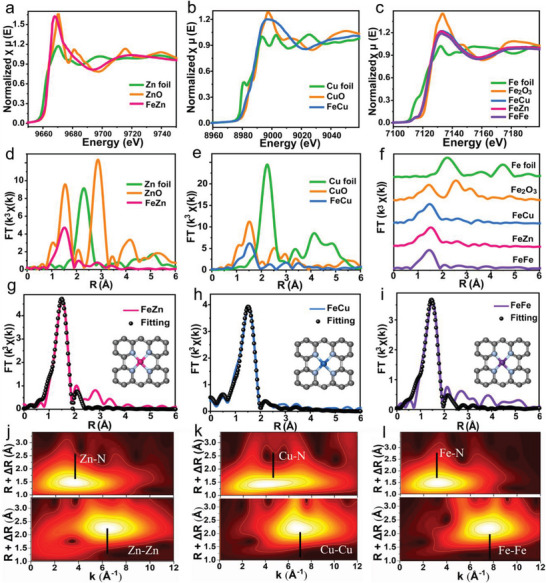
Structural characterization by X‐ray absorption spectroscopy. XANES spectra at a) Zn K‐edge of FeZn‐NC, b) Cu K‐edge of FeCu‐NC and c) Fe K‐edge of FeFe‐NC, in reference of metallic oxides and foils. Corresponding FT‐EXAFS spectra of d) FeZn‐NC, e) FeCu‐NC, and f) FeFe‐NC. Fitting curves at R space of g) FeZn‐NC, h) FeCu‐NC and (i) FeFe‐NC, the inset represents the structural model of M‐N_4_. WT‐EXAFS spectra at the corresponding K‐edge of j) FeZn‐NC, k) FeCu‐NC, and l) FeFe‐NC.

Furthermore, the FeZn‐NC possessed a similar near‐edge absorption with ZnO in Zn K‐edge XANES, indicating that the average valence state of Zn species was Zn^2+^ (**Figure** **3**a). The near edge and white line features in the Cu K‐edge profile were presented between Cu foil and CuO (Figure 3b), suggesting the oxidation states of Cu species in the FeCu‐NC. In addition, the Fe K‐edge XANES of FeZn‐NC, FeCu‐NC and FeFe‐NC was close to that of Fe_2_O_3_ (Figure 3c). Interestingly, compared to FeFe‐NC, the white line features of the Fe K‐edge spectra in FeZn‐NC and FeCu‐NC exhibited a slightly negative shift, implying lower oxidation state for Fe species, which is consistent with the result of XPS analysis. In fourier‐transform EXAFS (FT‐EXAFS) spectra, the main peak located at around 1.50 Å for FeZn‐NC, FeCu‐NC and FeFe‐NC, which was attributed to the scattering path of metal‐nitrogen (Figure 3d–f). Remarkably, the comparison with respective metallic foils verified no homonuclear Zn—Zn, Cu—Cu, or Fe—Fe bonds in FeZn‐NC and FeCu‐NC, while an additional small peak can be found in the range of 2.0 to 2.5 Å, which may be caused by heteronuclear Fe‐M.^[^ ^38^, ^39^ ^]^ The FT–EXAFS curve fitting was conducted to obtain the quantitative structural parameters of the FeM‐NC and metallic foils (Figure 3g–i; Figure S17–S20, Supporting Information), and their corresponding parameters were displayed in Table S3 (Supporting Information). The fitting results clearly confirmed that the coordination numbers of Zn‐N, Cu‐N and Fe‐N were 3.7 ± 0.3, 4.3 ± 0.2 and 4.1 ± 0.4, respectively, and their corresponding M—N bond length were 2.02, 1.95 and 2.03 Å. These results suggested that the metallic atoms in FeM‐NC were dominated by the M—N_4_ environment (Table S3, Supporting Information), which can be further investigated by the analyses of wavelet transform (WT)‐EXAFS spectra. In Figure 3j–l and Figure S21 (Supporting Information), the WT contour plots of FeZn‐NC, FeCu‐NC and FeFe‐NC exhibited an intensity maximum at around 3.9 Å^−1^, corresponding to the M—N coordination in FeM‐NC. In addition, compared with metallic foil references, the intensity maximum at ≈7.5 Å^−1^ ascribed to homonuclear M—M or Fe‐Fe coordination cannot be observed for all the samples. The above data further identified the isolated features of Fe and M species in FeM‐NC samples.

### 2.4 Electrocatalytic ORR and OER Activities

**Figure 4:**
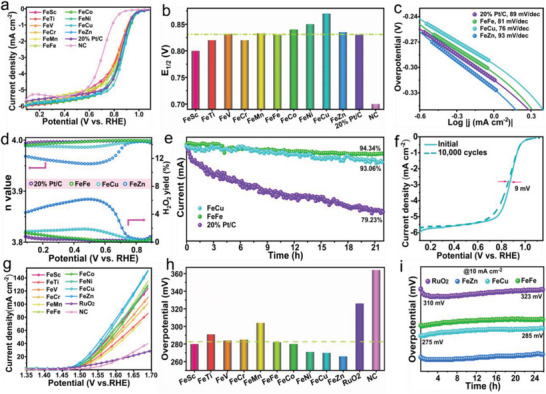
Bifunctional ORR and OER performances of FeM‐NC. a) ORR polarization curves and b) correpsonding half‐wave potential E_1/2_ for the as‐obtained FeM‐NC in 0.1 m KOH solution. c) Tafel plots and d) electron transfer number of FeFe‐NC, FeCu‐NC and FeZn‐NC. e) ORR stability test by chronoamperometry and f) LSV curves before and after 10 000 cycles of ADT for FeCu‐NC. g) OER polarization curves and h) corresponding OER overpotentials of the ten representative FeM‐NC in 1.0 m KOH solution. i) OER stability measurements of FeFe‐NC, FeCu‐NC and FeZn‐NC by chronopotentiometry at 10 mA cm^−2^.

To obtain the ORR performance of the as‐obtained catalysts, the linear sweep voltammetry (LSV) curves were recorded in **Figure** **4**a, in which the FeCu‐NC exhibited the best ORR activity with a limiting current density close to 6.0 mA cm^−2^ among all the measured FeM‐NC catalysts. In detail, the FeFe‐NC showed a half‐wave potential (E_1/2_) of 0.830 V and was comparable to that of Pt/C, while the NC without any metallic atom exhibits very poor activity (Figure 4b). Compared to FeFe‐NC, the early transition metals (Sc, Ti, V and Cr) delivered weak enhancement for the ORR activity, while the late transition metals (Co, Ni, Cu and Zn) exhibited obviously enhanced ORR activity. Interestingly, the regulable ORR activity of the late transition metals in FeM‐NC presented an increasing tendency except for Zn. The high ORR activity of FeCu‐NC was further confirmed by the low Tafel slope (76 mVdec^−1^, Figure 4c). The ring and disk currents were studied for all the catalysts (Figure S22, Supporting Information). Correspondingly, the FeNi‐NC and FeCu‐NC showed a dominate four‐electron pathway, and also delivered low peroxide (H_2_O_2_) yield less than 1.9% (Figure 4d). The stability of FeFe‐NC and FeCu‐NC were evaluated by chronoamperometry (i–t) and accelerated durability test (ADT). After running i–t test for 80 000s at 0.70 V, the FeFe‐NC and FeCu‐NC remained 94.3% and 93.0% of the pristine catalytic activity, while commercial 20% Pt/C revealed a high current attenuation of 20.7% (Figure 4e). In addition, a negligible decrease for the E_1/2_ and limiting current density could be obtained in FeCu‐NC after operating 10 000 cycles of ADT, which confirmed the high stability of FeCu‐NC catalyst (Figure 4f; Figure S23, Supporting Information).

BSACs are capable of achieving regulable electrocatalytic performance and multifunctional features due to the multiple active sites.^[^ ^40^, ^41^ ^]^ As expected, all the FeM‐NC were endowed with higher OER activity compared to those of RuO_2_ and NC (Figure 4g). Among the FeM‐NC catalysts, FeZn‐NC exhibits the lowest overpotential of 266 mV toward OER at 10 mA cm^−2^ (Figure 4h). Compared to FeFe‐NC, the early‐transition metals (Sc, Ti, V and Cr) showed little effects on the OER activity of FeM‐NC catalysts, whereas the late‐transition metals (Co,Ni,Cu,and Zn) greatly promoted their OER activity and tended an increasing enhancement, agrees with the ORR data. Notably, the FeCu‐NC also exhibited the highest OER activity, indicating its excellent bifunctional performance. The experimental results seem to be a slight deviation from the DFT results, which suggests FeNi‐NC should exhibit the best bifunctional activity, and the reasons are probably due to the possible defect effects in FeM‐NC and the ideal model in FeM‐N_6_C.^[^ ^42^, ^43^ ^]^ In fact, the bifunctional performance of FeNi‐NC was slightly lower than that FeCu‐NC, which are both near the peak of the volcano. Furthermore, the FeCu‐NC also verified the high performance at large current densities (50 and 100 mA cm^−2^), compared to FeCo‐NC and FeNi‐NC (Figure S24, Supporting Information). Finally, the stability of FeFe‐NC, FeCu‐NC, and FeZn‐NC catalysts were conducted for 24 h at 10 mA cm^−2^, and no obvious attenuation were observed (Figure 4i), suggesting their superior stability toward OER. After testing ORR and OER stability, a negligible decreasing uniformity and a slight aggregation for the isolated atoms were found in the FeCu‐NC, which also suggests its superior stability (Figure S25, Supporting Information).

### 2.5 Rechargeable Zn–Air Battery Test

**Figure 5:**
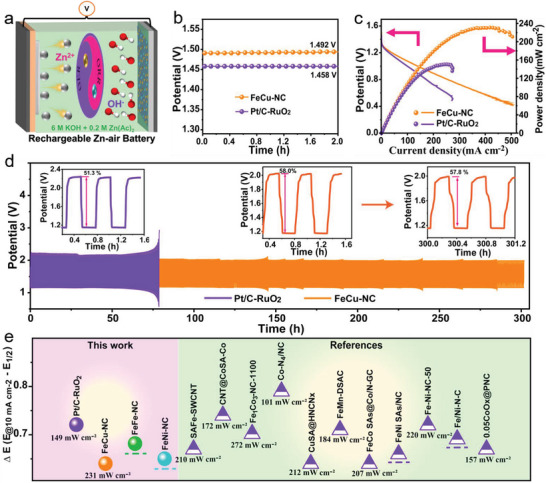
The performance of rechargeable zinc–air battery system of FeCu‐NC. a) Schematic of rechargeable Zn–air battery. b) Open circuit potentials, c) discharge polarization curves and power density plots, d) charge–discharge stabilities at the current density of 5.0 mA cm^−2^. e) Comparison of the bifunctional OER and ORR activities of FeCu‐NC with representative catalysts in 0.1 m or 1.0 m KOH solution, and their corresponding maximum power density.

Based on the low potential gap between ORR and OER of FeCu‐NC, the rechargeable Zn–Air battery (ZAB) was assembled (**Figure** **5**a), and Pt/C‐RuO_2_ with a mass ratio of 1:1 was used for comparison. The FeCu‐NC based ZAB exhibited an open circuit potential of 1.492 V (Figure 5b), which was higher than that of Pt/C‐RuO_2_ (1.458 V). The maximum power density was reached 231 mW cm^−2^ (Figure 5c), which was distinctly higher than that of Pt/C‐RuO_2_ (149 mW cm^−2^). The initial discharge and charge voltages of the FeCu‐NC based ZAB were 1.17 and 2.02 V, respectively, inducing a high round‐trip efficiency of 58.0% (Figure 5d). After continuous discharge and charge cycling for 300 h at 5 mA cm^−2^, the voltage gap of the FeCu‐NC based ZAB remained to be stable, corresponding to a negligible decrease from 58.0% to 57.8% in round‐trip efficiency. In contrast, a higher voltage gap (Δ*E*) of 1.09 V can be found in the Pt/C‐RuO_2_ based ZAB, suggesting the lower round‐trip efficiency (inset of Figure 5d), confirming the high ZAB performance and long‐term stability of the FeCu‐NC catalyst. Finally, compared with previously reported SACs, FeCu‐NC shows promising bifunctional activity and ZAB performance (Figure 5e and Table S4, Supporting Information).

## 3 Conclusion

In summary, we first employed the DFT calculations to reveal the synergistic effects between Fe and another 3d transition metals of FeM BSACs toward bifunctional ORR and OER, and a notable volcano relationship was established between the performance and the adsorption free energy. Notably, the late transition metals (Co, Ni, Cu, and Zn) can more efficiently optimize the adsorption free energy on Fe sites for both the ORR and OER in comparison to the early transition metals (Sc, Ti, V, and Cr), and the regulable tends of the bifunctional performance for FeM BSACs were further verified by the experimental data. The FeCu‐NC catalyst, which is located near the peak of the volcano curves, exhibits high ORR and OER activity, as well as the lowest overpotential gap for bifunctional catalysis. This work not only demonstrated how to rationally design effective FeM BSACs toward bifunctional ORR and OER, but also established a new method for the screening of new catalysts for the bifunctional oxygen electrocatalysts.

## 4 Experimental Section

### Materials

All of the chemicals mentioned in the synthesis steps were used directly without further treatment. Melamine (≥99%), metal chloride (>99.9%) and potassium hydroxide (99.99%) were purchased from Shanghai Aladdin Biochemical Technology Co., Ltd., China. The hydrochloric acid (≈37 wt.%) and zinc acetate (99.5%) were bought from Sinopharm Chemical Reagent Co., Ltd., China. The Zn–air battery accessories, including carbon paper, zinc plates and 5 wt.% of nafion solution, etc., were produced from Shanghai Hesen Electrical Co., Ltd., China.

### Preparation of M‐C_3_N_4_ as Precursors

A series of M‐C_3_N_4_ (M = Sc, Ti, V, Cr, Mn, Fe, Co, Ni, Cu and Zn) were synthesized as metallic precursors. Typically, 0.33 mol of the corresponding metal chloride was dispersed into 150 mL of hydrochloric acid (4.80 wt.%) containing 9.0 g of melamine under stirring for 10 min, respectively. After subsequent ultrasound for 1 h, these emulsion solutions were evaporated to afford solid powder with the uniformly dispersed metals. Then, the as‐obtained solid powder was pyrolyzed at 550 °C for 2 h under a heating rate of 5 °C min^−1^. The final product was ground and marked as M‐C_3_N_4_ for further use.

### Preparation of Fe‐Based Binary Single‐Atom Electrocatalysts

The Fe‐based binary single‐atom electrocatalysts were prepared by calcining the substances of polydopamine coating M‐C_3_N_4_ and Fe‐C_3_N_4_. In detail, 1.0 g of a mixture containing M‐C_3_N_4_ and Fe‐C_3_N_4_ with a mass ratio of 1:1 was dispersed into 33 mL of Tris‐HCl buffer solution (pH = 8.8) under ultrasound for 1 h. Then, 0.7 g of dopamine hydrochloride was added into the buffer solution. After polymerization for 24 h, the polydopamine‐coated Fe‐C_3_N_4_ and M‐C_3_N_4_ powder was collected by filtration and washed with ultrapure water. The resulting product was dried at 60 °C and calcined at 800 °C for 2 h under Ar atmosphere to obtain dual‐metal Fe and M atoms supported on nitrogen‐doped carbon (labeled as FeM‐NC, M = Sc, Ti, V, Cr, Mn, Fe, Co, Ni, Cu and Zn). For comparation, nitrogen‐doped carbon was synthesized by the similar process without doping any metal.

### Characterizations

Material characterization methods, including XRD, XPS, Raman, N_2_ adsorption–desorption isotherms, ICP‐MS, TEM and EDS mapping, AC‐STEM, XAS, and DFT calculations were described in the “Supporting Information”. Electrochemical measurements were also provided in the “Supporting Information”, including LSV, electron transfer number, chronoamperometry, chronopotentiometry, power density and charging–discharging stability in Zn–air batteries, etc.

### Statistical Analysis

Pre‐processing of the data was carried out in Excel and the final data were statistically analyzed and plotted using Origin software. Electrochemical test data were converted with reference to reversible hydrogen electrodes, and multiple measurements were carried out to present the accurate results.

## Conflict of Interest

The authors declare no conflict of interest.

## Supporting information

Supporting Information

## Data Availability

The data that support the findings of this study are available from the corresponding author upon reasonable request.
