# Supplementary material for: Understanding the Bifunctional Trends of Fe‐Based Binary Single‐Atom Catalysts
Source: Adv Sci (Weinh). 2023 Jun 21;10(24):2301566. doi: 10.1002/advs.202301566 (PMC10460889; doi:10.1002/advs.202301566)
Supplement: Supplementary file 1 — Supporting Information [file ADVS-10-2301566-s001.pdf]

ADVANCED SCIENCE
Open Access

Supporting Information

for *Adv. Sci.*, DOI 10.1002/advs.202301566

Understanding the Bifunctional Trends of Fe-Based Binary Single-Atom Catalysts

Ruisong Li, Peng Rao, Daoxiong Wu, Jing Li, Peilin Deng*, Zhengpei Miao and Xinlong Tian**

Supporting information for

Understanding the bifunctional trends of Fe-based binary single-atom catalysts

Ruisong Li, Peng Rao, Daoxiong Wu*, Jing Li, Peilin Deng*, Zhengpei Miao, and Xinlong Tian*

State Key Laboratory of Marine Resource Utilization in South China Sea, Hainan Provincial Key Lab of Fine Chemistry, School of Chemical Engineering and Technology, Hainan University, Haikou 570228, China

***Corresponding author**: *daoxiong@hainanu.edu.cn (D. Wu); dengpeilin@hainanu.edu.cn (P. Deng); tianxl@hainanu.edu.cn (X. Tian)*

Experimental Methods

Materials Characterization

The phase structures of the catalysts were characterized by X-ray powder diffractometer (XRD) patterns on Bruker D8 Advance X-ray diffractometer. Raman spectra were measured by a WiTech alpha300R confocal Raman microscopic system with a 532 nm excitation laser. The concentrations of metal in catalysts were determined by inductively coupled plasma mass spectrometry (ICP-MS) on Agilent 7700s. The specific surface area and pore size distribution were evaluated using Barrett-Emmett-Teller calculation according to N_2 adsorption-desorption isotherm on Mike ASAP2460. Transmission electron microscopy (TEM) images and energy disperse spectroscopy (EDS) elemental mapping were carried out on Talos F200X G2. Aberration-corrected scanning transmission electron microscopy (AC-STEM) images were captured on Nion Ultra STEM U100 with a Gatan Enfina electron energy loss spectrometer. X-ray photoelectron spectroscopy (XPS) measurements were recorded on Thermo ESCALAB 250XI. The X-ray absorption spectroscopy (XAS) analysis including X-ray absorption near edge structure (XANES) and extended X-ray absorption fine structure (EXAFS) at Fe, Cu and Zn K-edge were measured at the beamline 1W1B station of Shanghai Synchrotron Radiation Facility (BSRF), China.

Electrochemical measurements

The electrochemical measurements were performed on a Gamry electrochemical workstation with a three-electrode system using saturated Hg/HgO electrode and carbon rod as the counter electrode and reference electrode, respectively. For ORR activity, the ink could be obtained by blending 5 mg of catalyst, 50 μL of 5 wt.% Nafion and 950 μL of isopropanol under ultrasound for 30 minutes. Then, 8 μL of the ink was dropped on glass carbon electrode (rotating disk electrode, RDE, Φ = 5 mm) and measured O_2-saturated 0.1 M KOH solution. Meanwhile, the OER activity of catalyst was tested on L-type glass carbon electrode (Φ = 5 mm). To fastness catalyst on the electrode surface during OER testing, the amount of Nafion was increased to 100 μL. And the electrochemical test was carried out in 1.0 M KOH solution. All

the potentials were recorded relative to the reversible hydrogen electrode (vs. RHE).

Linear sweep voltammetry (LSV) measurements were carried out on RDE at a sweep rate of 5 mV s^{-1} under 1600 rpm in O_2-saturated 0.1 M KOH solution. The electron kinetic current density (J_k) can be calculated from the Koutecky-Levich (K-L) equation:

$$1/J = 1/J_L + 1/J_K$$

Where J is the measured current density, J_K represents the kinetic current density, J_L represents the obtained diffusion-limited current density. The number of transfer electrons (n) and hydrogen peroxide yield were determined on rotating ring disk electrode (RRDE) by the following equation:

$$H_2O_2\ (\%) = 200 \times (I_r/N) / (I_d + I_r/N)$$

$$n = 4 \times I_d / (I_r/N + I_d)$$

Where I_d and I_r represent the disk current and ring current, respectively. N is the current collection efficiency of Pt ring towards intermediate reaction and defined as 0.37.

To access the performance of electrocatalyst in practical application, the flexible Zn-air batteries were assembled. In this system, a Zinc foil was used as anode and the carbon paper coated with 1 mg cm^{-2} of catalyst ink was regarded as air cathode. The electrolyte contained 6 M KOH and 0.2 M $Zn(Ac)_2$. All data were collected from the as-prepared battery with a Land CT2001A system and a Gamry electrochemical workstation.

XAS analysis

Data reduction, data analysis, and EXAFS fitting were performed with the Athena and Artemis software packages.[1] The energy calibration of the sample was conducted through standard Fe foil, Cu foil and Zn foil, which as a reference was simultaneously measured. A linear function was subtracted from the pre-edge region, then the edge jump was normalized using Athena software. The $\chi(k)$ data were isolated by subtracting a smooth, three-stage polynomial approximating the absorption background of an isolated atom. The k^3-weighted $\chi(k)$ data were Fourier transformed after applying a Hanning window function ($\Delta k = 0.5$).

For EXAFS modeling, the global amplitude EXAFS (CN, R, S_0^2, σ^2 and ΔE_0) were obtained by nonlinear fitting, with least-squares refinement, of the EXAFS equation to the Fourier-transformed data in R-space, using Artemis software, EXAFS of the Fe foil, Cu foil and Zn foil are fitted and the obtained amplitude reduction factor S_0^2 value (0.783, 0.775 and 0.801) was set in the EXAFS analysis to determine the coordination numbers (CNs) in the Fe/Cu/Zn-N scattering path in sample. For Wavelet Transform analysis, the $\chi(k)$ exported from Athena was imported into the Hama Fortran code.[2] The parameters were listed as follow: R range, 1 - 3.5 Å, k range, 0 - 13.0 $Å^{-1}$ for sample (0-13.0 $Å^{-1}$ for foil and standards); and Morlet function with κ=10, σ=1 was used as the mother wavelet to provide.

DFT analysis

Spin-polarized density functional theory calculations were performed using the Perdew-Burke-Ernzerhof (PBE) functional[3] and the projector augmented wave (PAW) potential[4, 5] as implemented in the Vienna Ab Initio Simulation Package (VASP).[6, 7] Considering the potential strong correlation in *d* orbitals of 3d transition metal, the DFT + U method with effective U_{eff} value adopted from Zeng et al[8] was used in our calculations. An energy cutoff of 400 eV and a convergence criterion of 10^{-5} eV for self-consistent calculations was adopted. All structures were fully relaxed until the total force on each atom was less than 0.05 eV/ Å. The solvent effect was included by using the implicit solvation model as implemented in the VASPsol code.[9, 10] The thickness of the vacuum layer was large than 14 Å. Our single-atom catalyst models were built based on 6 × 6 × 1 graphene supercells. A Γ-centered 3 × 3 × 1 k-point was adopted. VASPKIT code[11] and VESTA software[12] were used for calculation pre-processing and post-processing.

The computational hydrogen electrode (CHE) model[13] was used in our calculations. The Gibbs free energy of molecules and ORR-related adsorbates was calculated by $G = E_{DFT} + ZPE - TS$, where E_{DFT}, ZPE, and S were the DFT energy, zero-point energy, and entropy, respectively, and temperature T was adopted as 298.15 K. The ORR involves four four-electron pathways:

a) $* + O_2 + H_2O + e^- \rightarrow OOH* + OH^-$, $\Delta G_1 = \Delta G_{OOH*} - 4.92$

b) $OOH* + e^- \rightarrow O* + OH^-$, $\Delta G_2 = \Delta G_{O*} - \Delta G_{OOH*}$

c) $O* + H_2O + e^- \rightarrow OH* + OH^-$, $\Delta G_3 = \Delta G_{OH*} - \Delta G_{O*}$

d) $OH* + e^- \rightarrow OH^- + *$, $\Delta G_4 = -\Delta G_{OH*}$

The ΔG_{OOH*}, ΔG_{O*}, and ΔG_{OH*} are calculated according to:

a) $\Delta G_{OOH*} = G(OOH*) - G(*) + 1.5\times G(H_2) - 2\times G(H_2O)$

b) $\Delta G_{O*} = G(O*) - G(*) + G(H_2) - G(H_2O)$

c) $\Delta G_{OH*} = G(OH*) - G(*) + 0.5\times G(H_2) - G(H_2O)$

The theoretical overpotential at equilibrium potential was determined according to $\eta_{ORR} = \max\{\Delta G_1, \Delta G_2, \Delta G_3, \Delta G_4\}/e + 1.23$.

OER is considered to be the inverse process of ORR, thus $\eta_{OERR} = \max\{-\Delta G_1, -\Delta G_2, -\Delta G_3, -\Delta G_4\}/e - 1.23$.

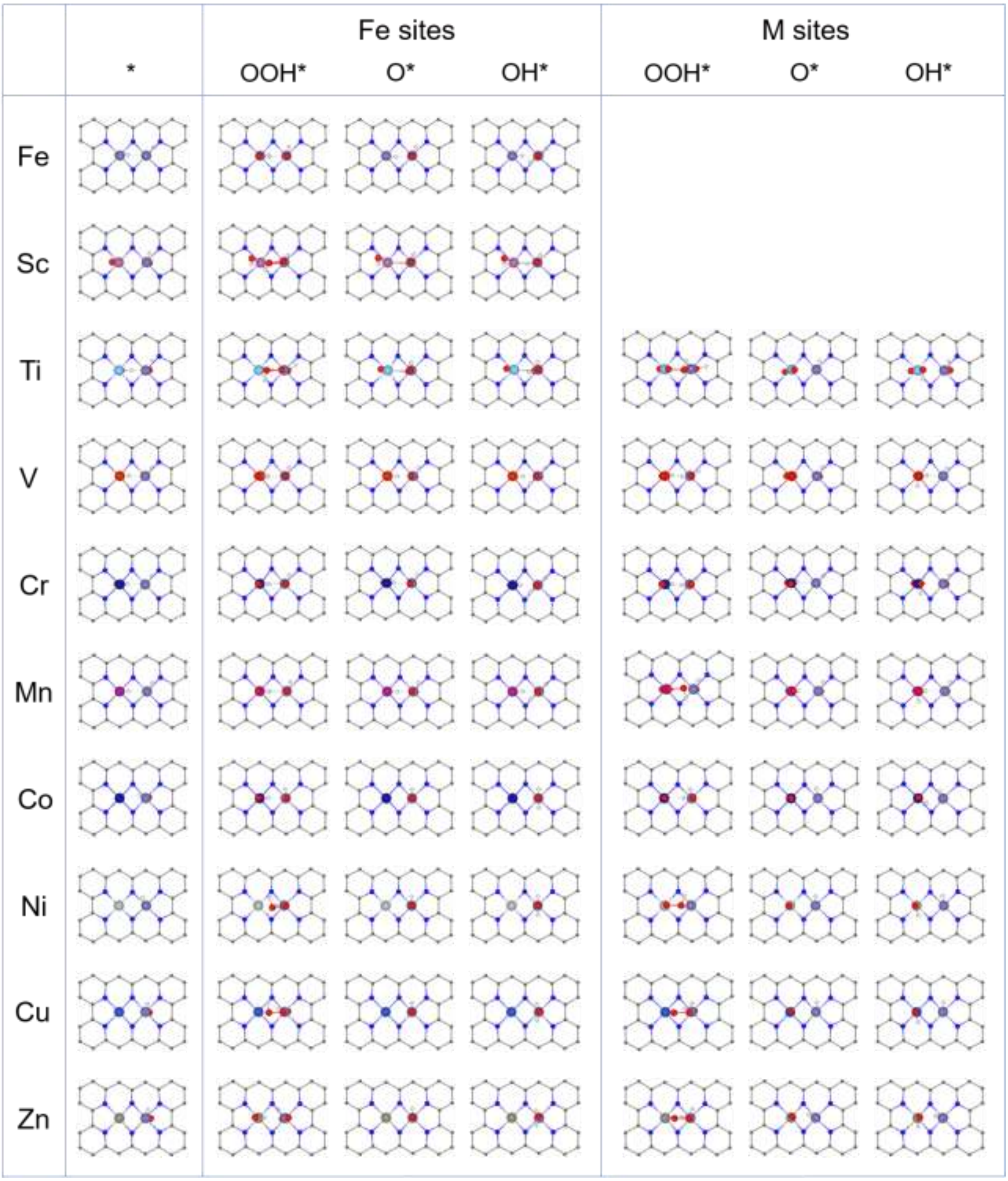

Figure S1. Atomic structures of pristine catalysts and adsorption conformations.

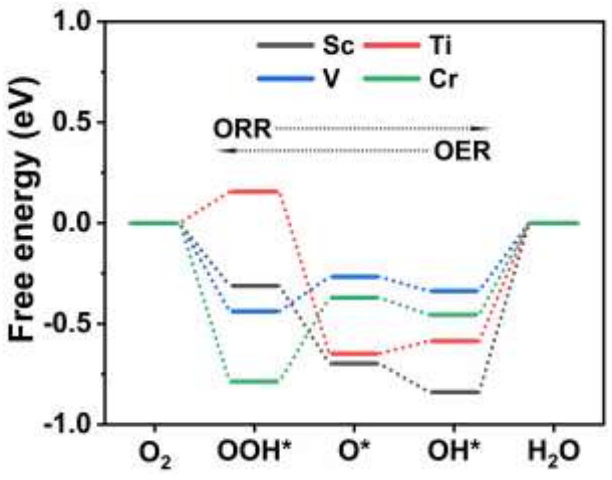

Figure S2. Calculated free energy diagram of Fe sites in FeSc-N_6C, FeTi-N_6C, FeV-N_6C and FeCr-N_6C.

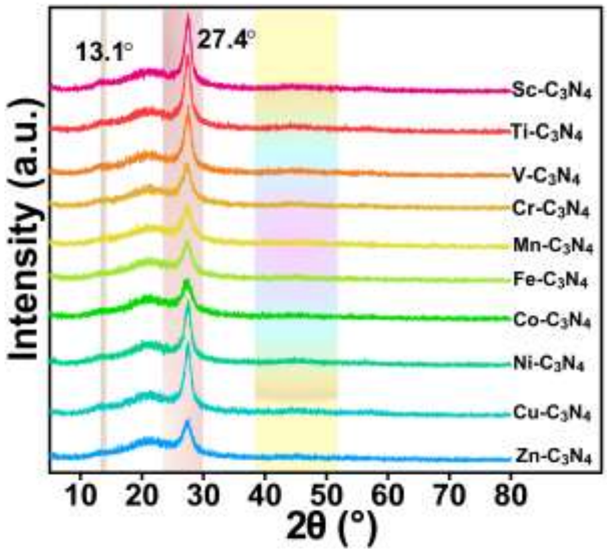

Figure S3. XRD patterns of M-C_3N_4 as metallic precursors.

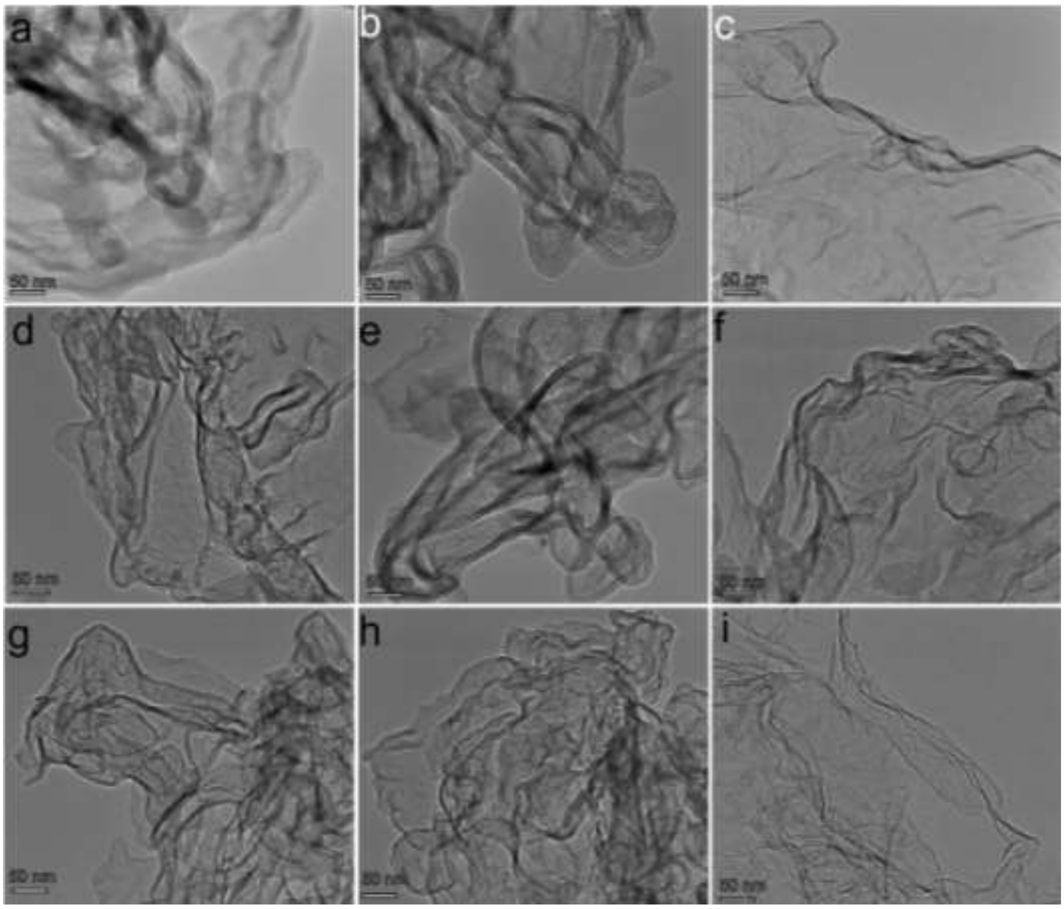

Figure S4. High-magnification TEM images of (a) FeSc-NC, (b) FeTi-NC, (c) FeV-NC, (d) FeCr-NC, (e) FeMn-NC, (f) FeFe-NC, (g) FeCo-NC, (h) FeNi-NC and (i) FeZn-NC.

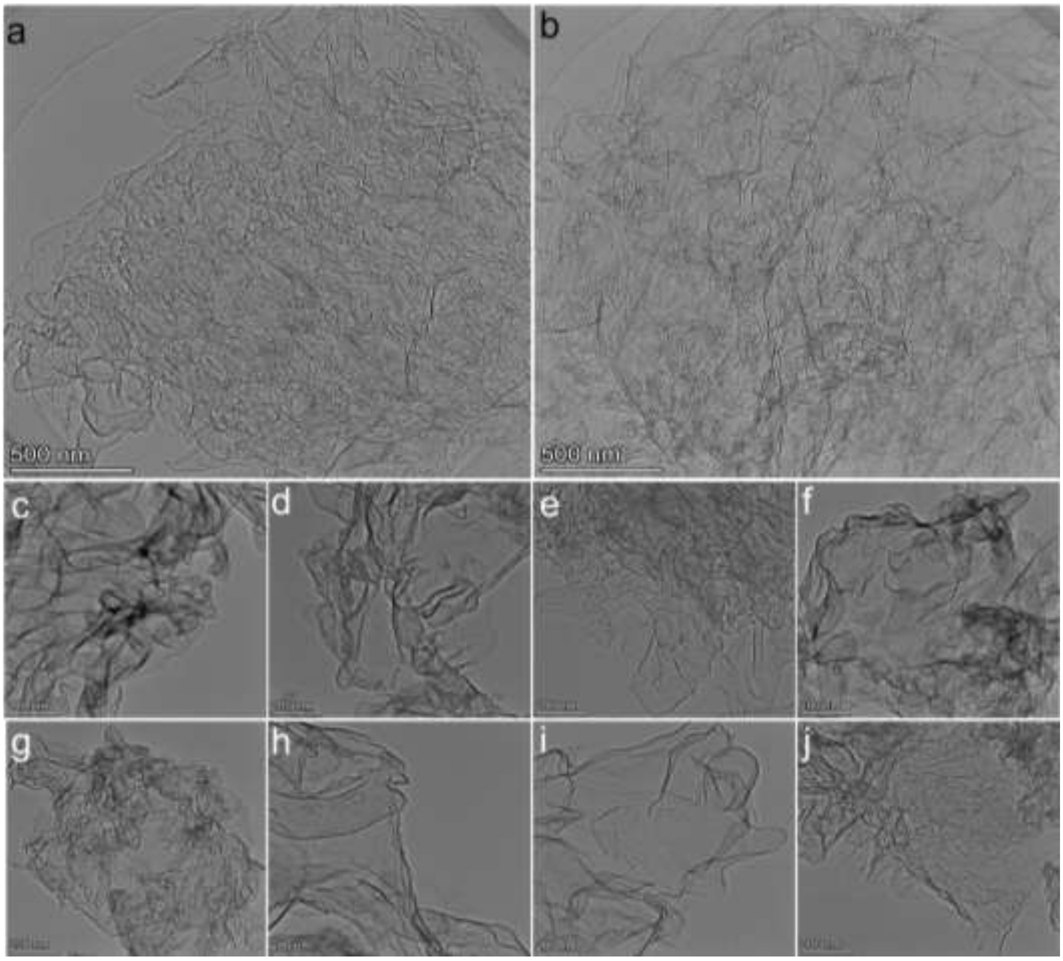

Figure S5. Low-magnification TEM images of (a) FeSc-NC, (b) FeTi-NC, (c) FeV-NC, (d) FeCr-NC, (e) FeMn-NC, (f) FeFe-NC, (g) FeCo-NC, (h) FeNi-NC, (i) FeCu-NC and (j) FeZn-NC.

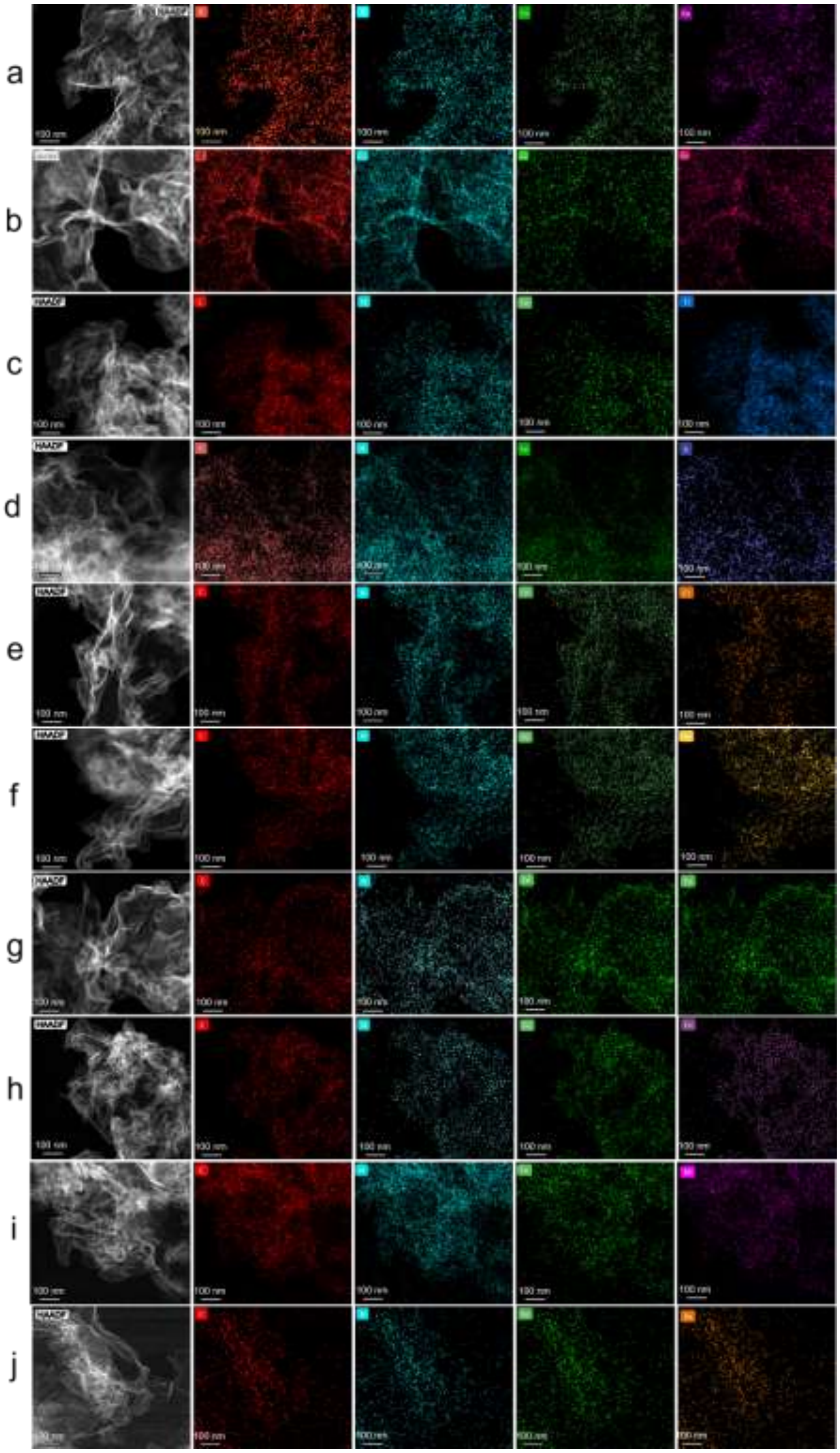

Figure S6. EDX mapping of (a) FeCu-NC, (b) FeSc-NC, (c) FeTi-NC, (d) FeV-NC, (e) FeCr-NC, (f) FeMn-NC, (g) FeFe-NC, (h) FeCo-NC, (i) FeNi-NC and (j) FeZn-NC.

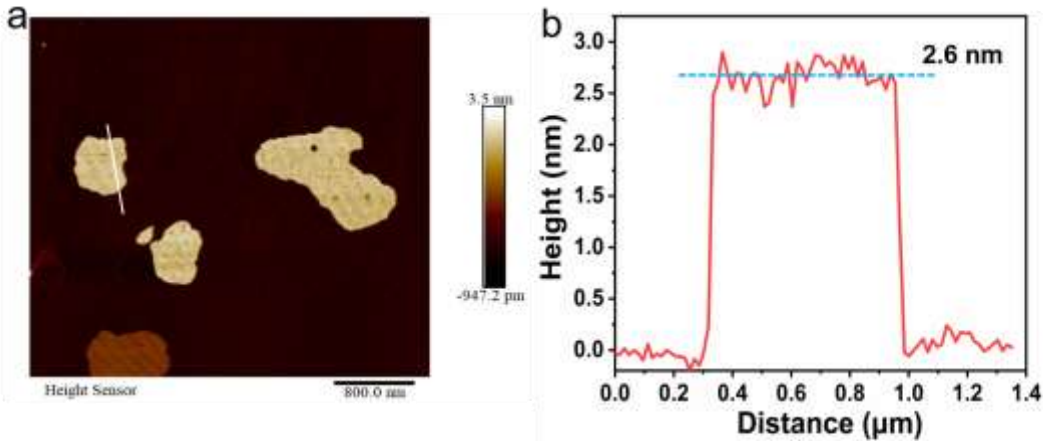

Figure S7. (a) Atomic force microscope image and (b) corresponding height profiles along the white line.

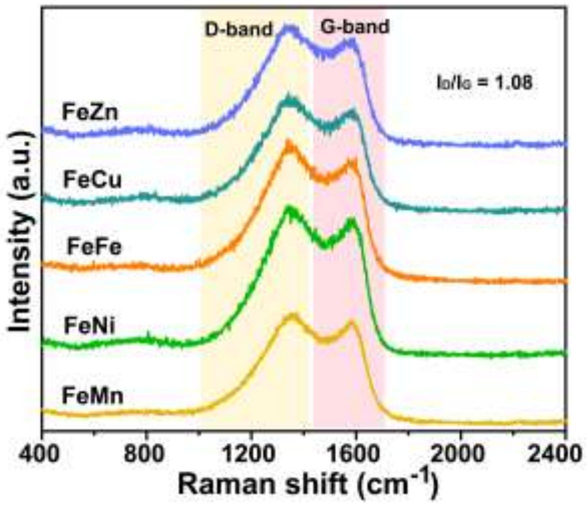

Figure S8. Raman spectra of FeMn-NC, FeNi-NC, FeFe-NC, FeCu-NC and FeZn-NC, and their corresponding I_D/I_G value is approximately 1.08.

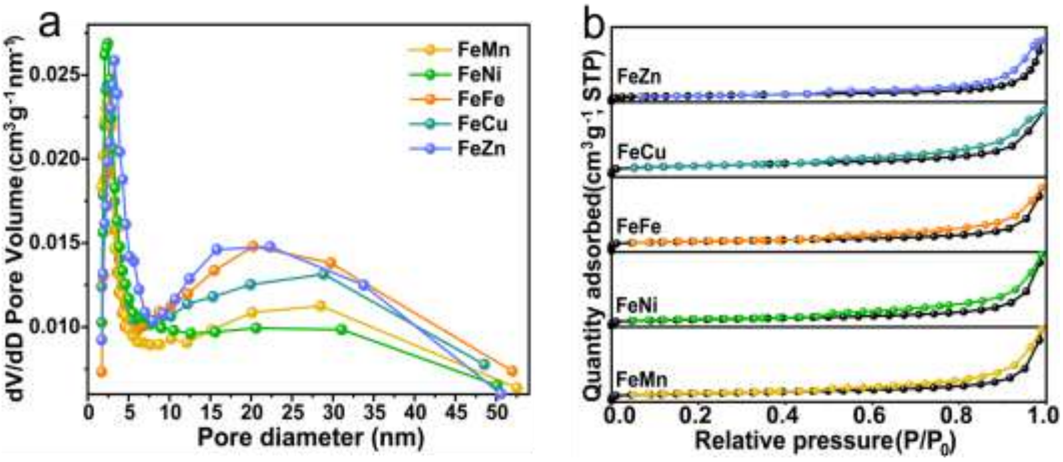

Figure S9. (a) Pore-size distribution curves and (b) N_2 adsorption-desorption isotherms of FeMn-NC, FeNi-NC, FeFe-NC, FeCu-NC and FeZn-NC.

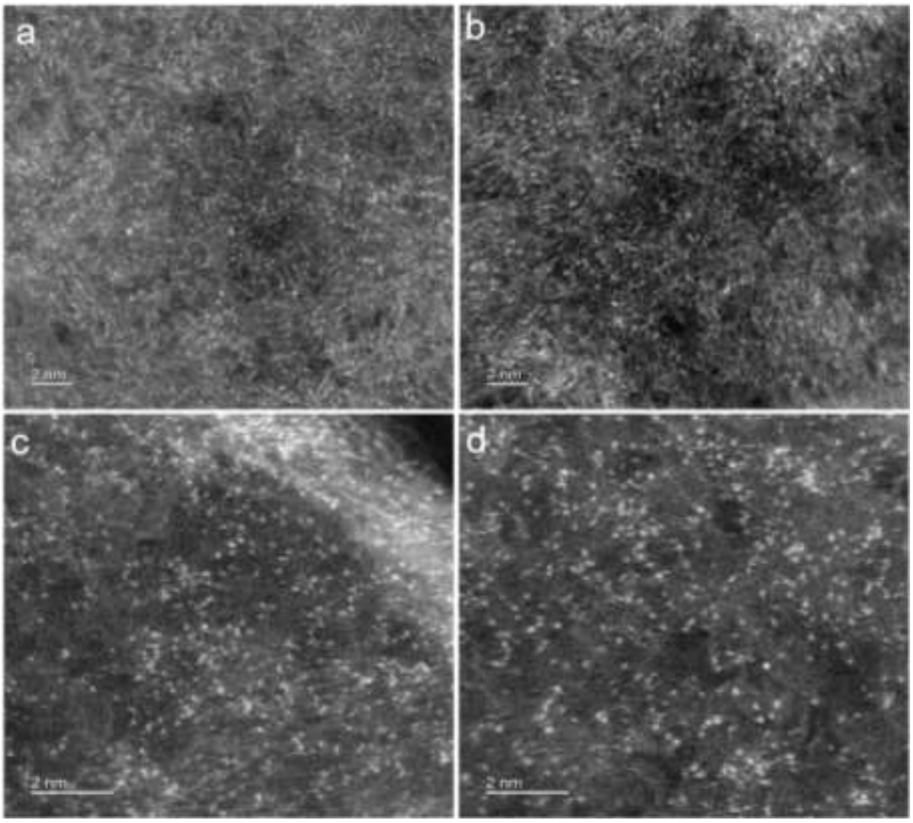

Figure S10. AC-STEM images of (a) FeMn-NC, (b) FeNi-NC, (c) FeFe-NC and (d) FeZn-NC.

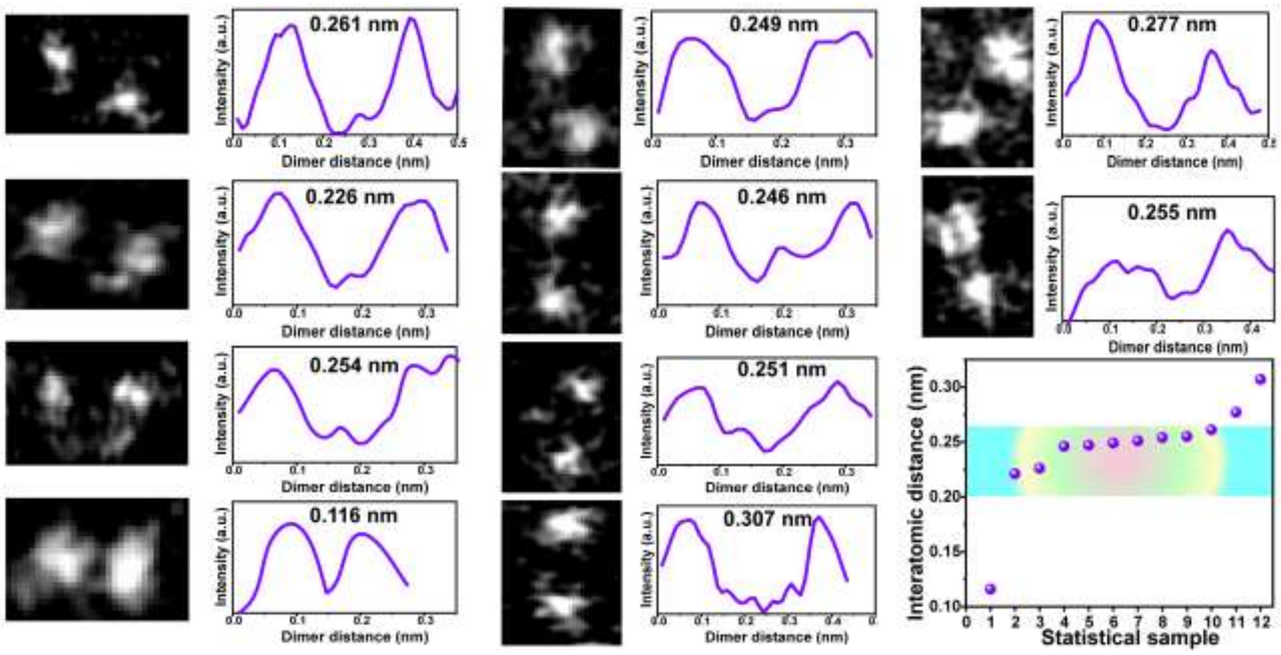

Figure S11. A dominant atomic spacing from 0.226 nm to 0.255 nm in FeCu-NC by simple statistics.

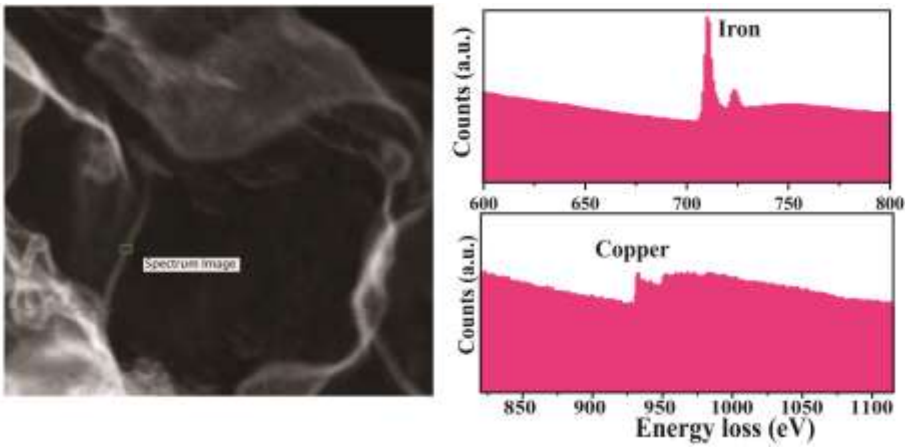

Figure S12. Electron energy loss spectroscopy of Fe and Cu atoms for FeCu-NC.

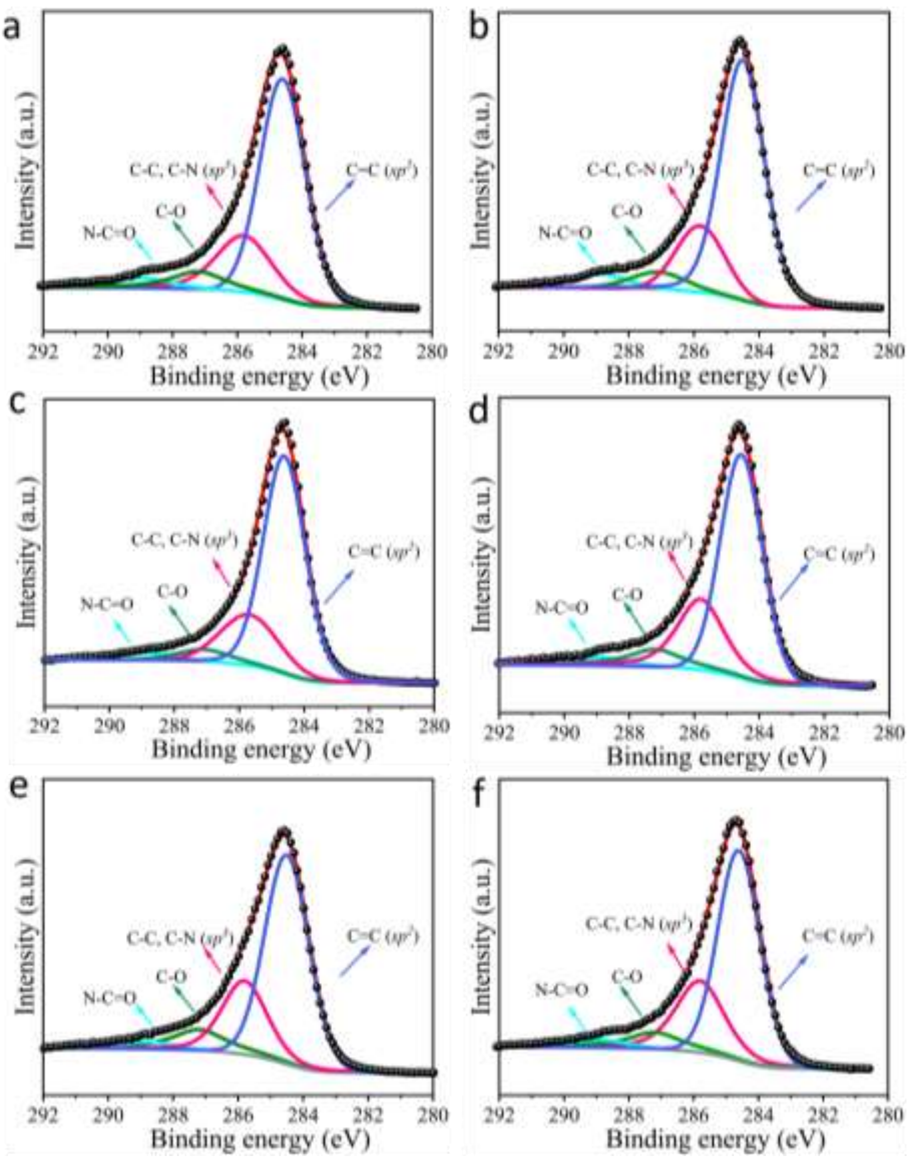

Figure S13. High-resolution C 1s XPS spectra of (a) FeMn-NC, (b) FeNi-NC, (c) FeFe-NC, (d) FeCu-NC, (e) FeZn-NC and (f) NC.

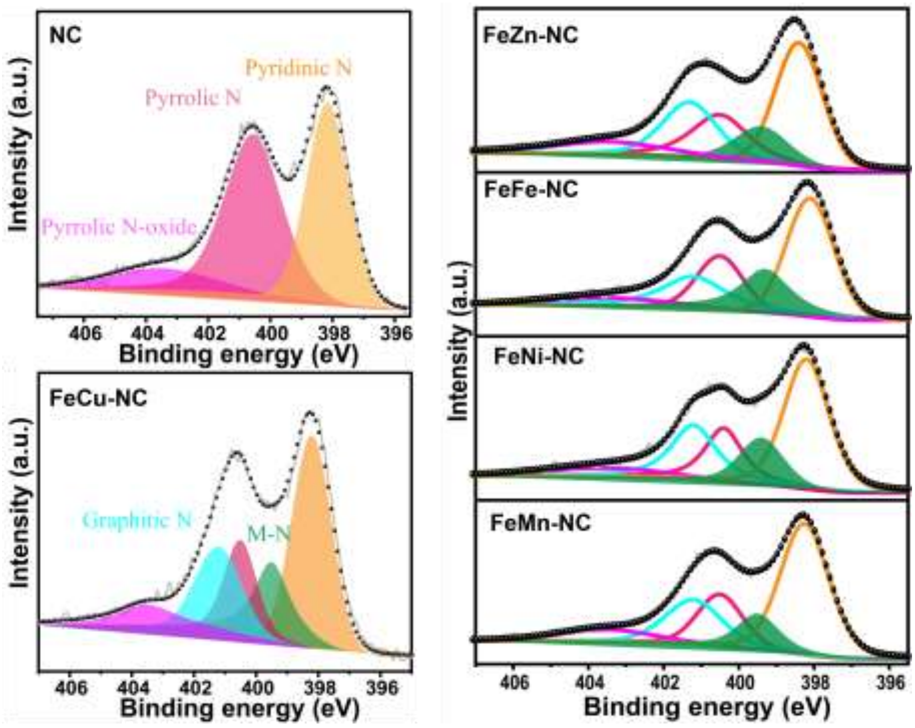

Figure S14. High-resolution N 1s XPS spectra of FeMn-NC, FeNi-NC, FeFe-NC, FeCu-NC, FeZn-NC and NC.

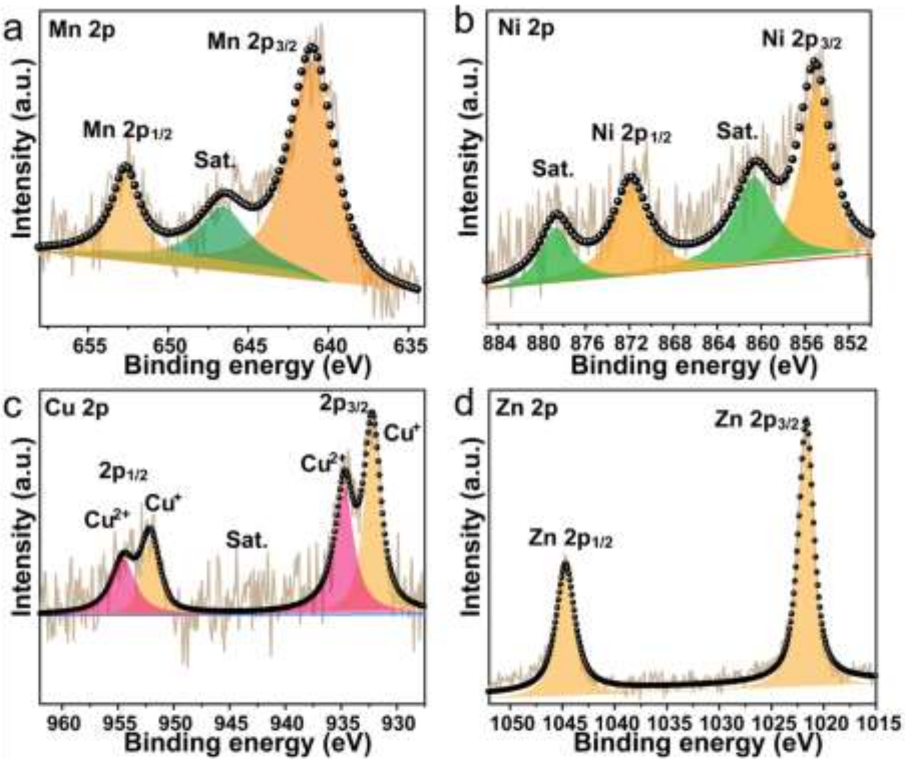

Figure S15. High-resolution M_{2p} XPS spectra of (a) FeMn-NC, (b) FeNi-NC, (c) FeCu-NC and (d) FeZn-NC.

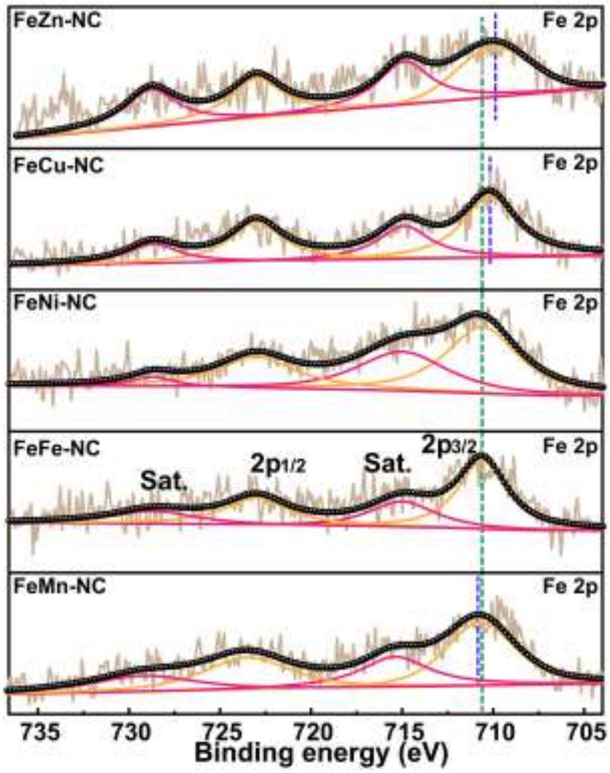

Figure S16. High-resolution Fe 2p XPS spectra of FeMn-NC, FeFe-NC, FeNi-NC, FeCu-NC and FeZn-NC.

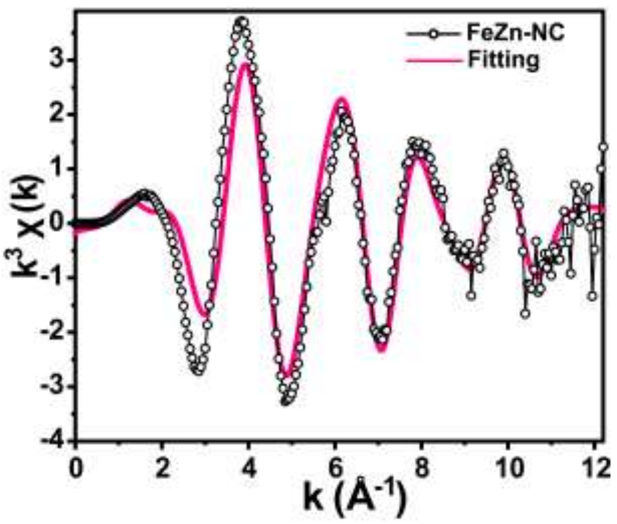

Figure S17. FT-EXAFS fitting curves at k space for Zn species in FeZn-NC.

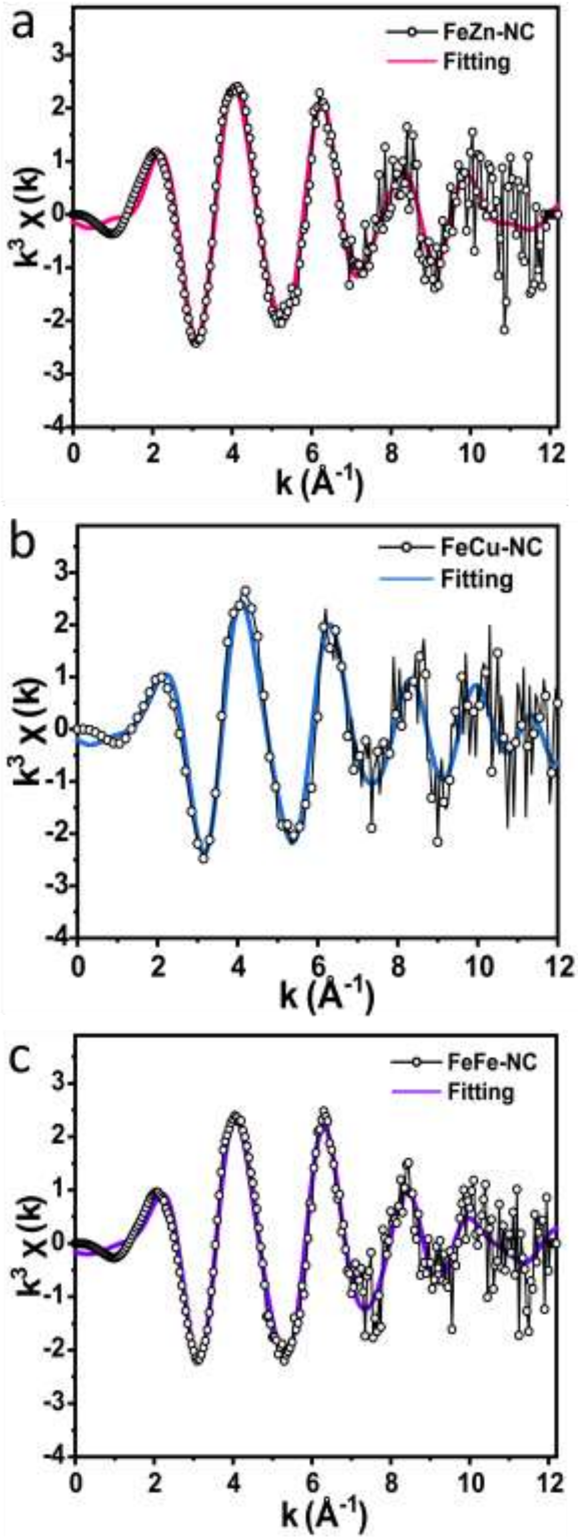

Figure S18. FT-EXAFS fitting curves at k space for Fe species in (a) FeZn-NC, (b) FeCu-NC and (c) FeFe-NC.

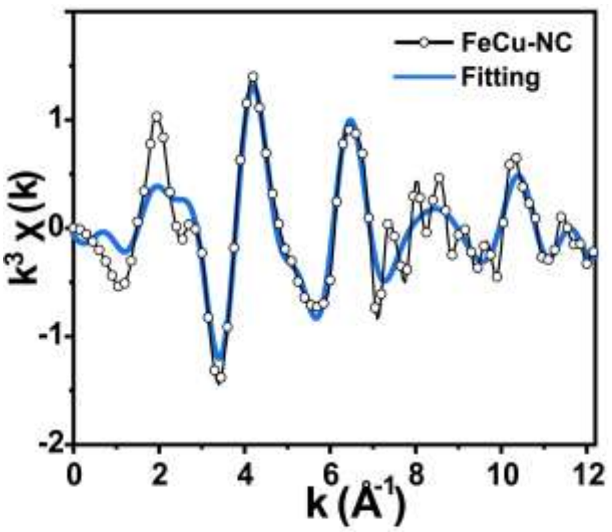

Figure S19. FT-EXAFS fitting curves at k space for Cu species in FeCu-NC.

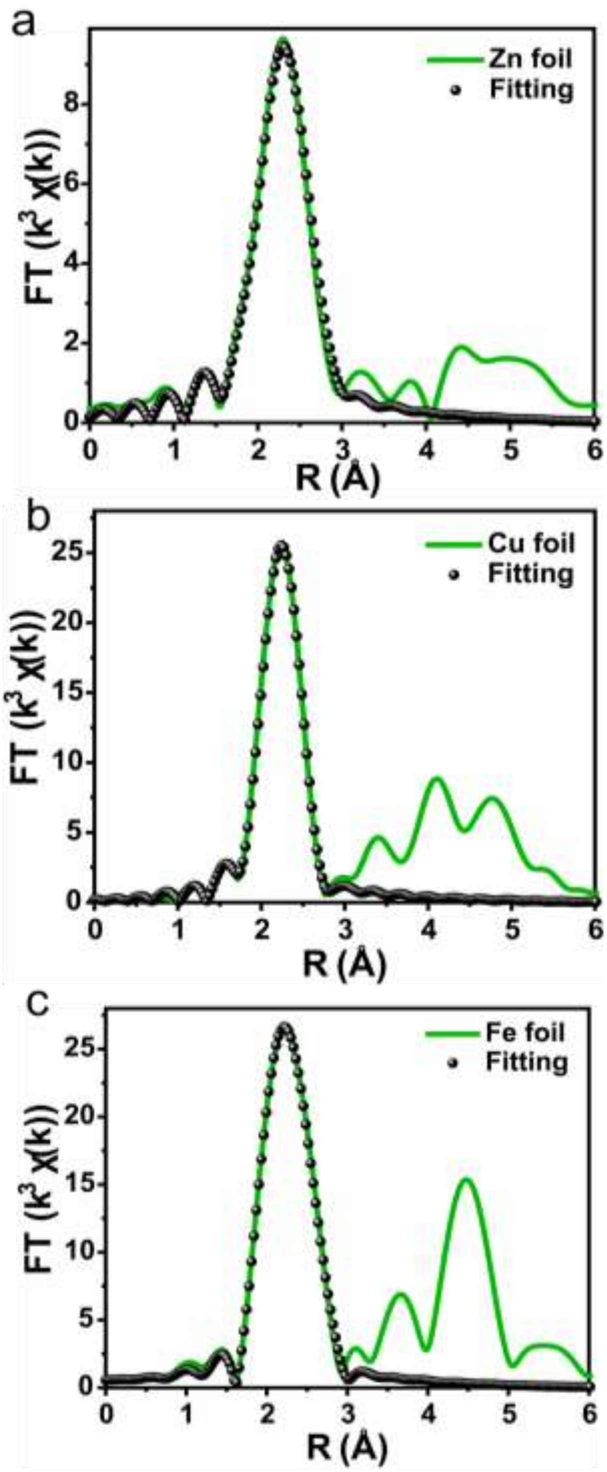

Figure S20. FT-EXAFS fitting curves at R space for (a) Zn foil, (b) Cu foil and (c) Fe foil.

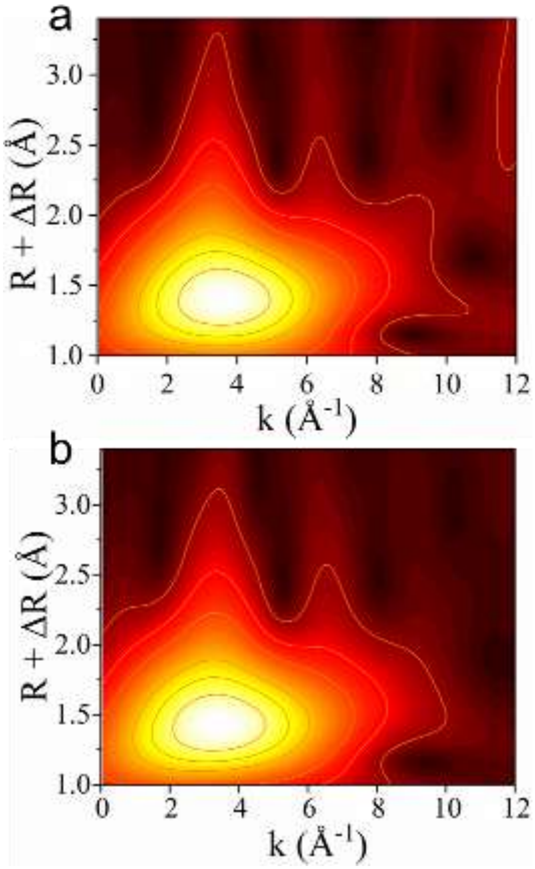

Figure S21. WT-EXAFS spectra for Fe species in (a) FeZn-NC and (b) FeCu-NC.

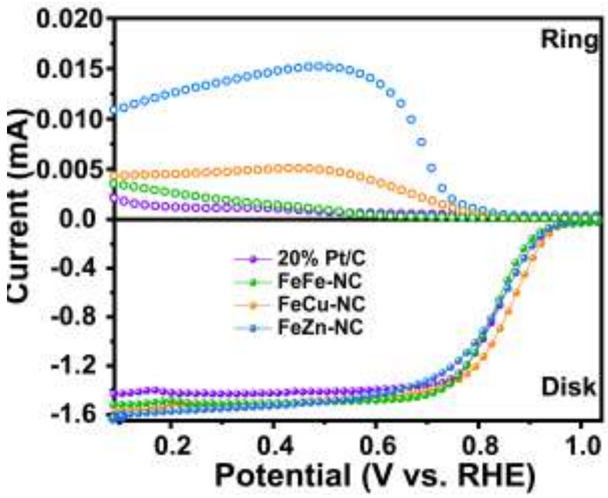

Figure S22. Experimental ring and disk currents of FeFe-NC, FeCu-NC and FeZn-NC based on RRDE test.

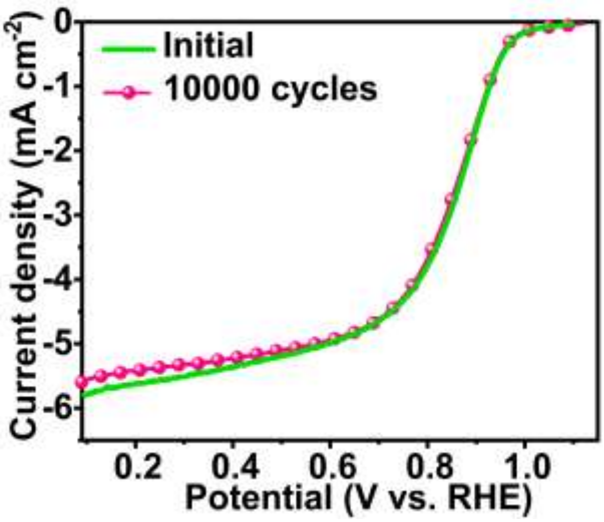

Figure S23. LSV curves of FeFe-NC before and after 10000 cycles of ADT.

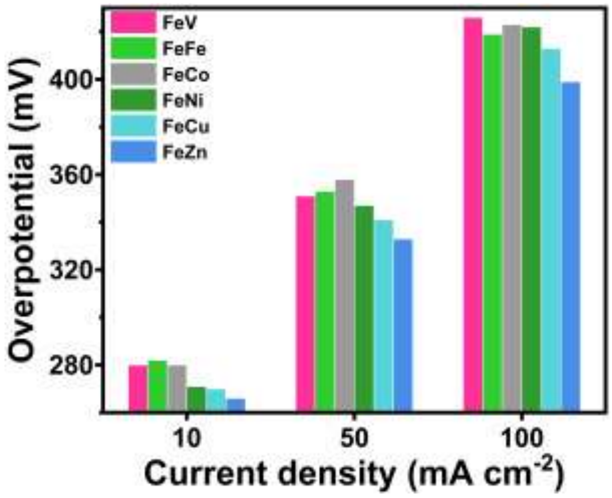

Figure S24. The corresponding overpotentials of FeV-NC, FeFe-NC, FeCo-NC, FeNi-NC, FeCu-NC and FeZn-NC at 10, 50 and 100 mA cm^{-2}, respectively.

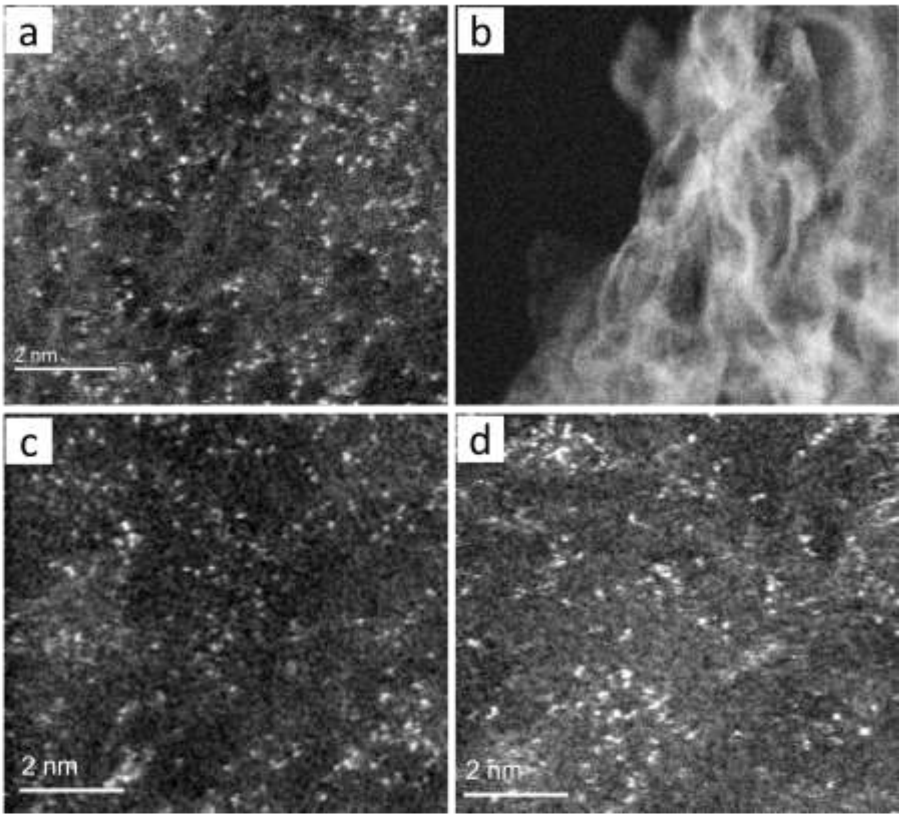

Figure S25. AC-STEM and HAADF images of the FeCu-NC for (a) primary, (b) and (c) testing ORR stability, (d) testing OER stability.

Table S1. Summary of theoretical ΔG_{OOH*} (eV), ΔG_{O*} (eV), ΔG_{OH*} (eV), $\Delta G_{O*} - \Delta G_{OH*}$ (eV), η_{ORR} (V), η_{OER} (V), and $\eta_{ORR} + \eta_{OER}$ (V).

		ΔG_{OOH*}	ΔG_{O*}	ΔG_{OH*}	$\Delta G_{O*} - \Delta G_{OH*}$	η_{ORR}	η_{OER}	$\eta_{ORR} + \eta_{OER}$
Fe sites	Sc	3.378	1.763	0.391	1.372	0.839	0.386	1.225
	Ti	3.846	1.812	0.644	1.168	0.586	0.803	1.389
	V	3.252	2.193	0.893	1.301	0.337	0.438	0.775
	Cr	3.071	2.200	0.813	1.387	0.417	0.619	1.036
	Mn	3.285	2.237	0.982	1.256	0.248	0.405	0.653
	Fe	3.184	2.200	0.791	1.409	0.439	0.506	0.945
	Co	3.453	2.220	0.896	1.323	0.334	0.237	0.570
	Ni	3.864	2.429	1.027	1.402	0.203	0.205	0.408
	Cu	4.127	2.675	1.283	1.392	0.437	0.222	0.659
	Zn	3.462	2.682	1.236	1.447	0.450	0.228	0.677
M sites	Ti	4.466	3.106	1.390	1.717	0.776	0.487	1.262
	V	2.952	1.770	1.001	0.769	0.461	0.738	1.199
	Cr	2.903	2.090	0.775	1.315	0.455	0.787	1.242
	Mn	3.858	2.615	0.697	1.918	0.533	0.688	1.221
	Co	3.641	2.542	0.762	1.781	0.468	0.551	1.019
	Ni	4.268	3.543	1.545	1.998	0.578	0.768	1.346
	Cu	4.686	4.002	1.603	2.398	0.996	1.168	2.164
	Zn	4.016	3.159	0.891	2.269	0.373	1.039	1.412

Table S2. Weight % of metal loading obtained from ICP-MS analysis, atomic % of elements obtained from XPS analysis, and specific surface area based on BET.

Sample	ICP-MS (wt.%)		XPS (at.%)			BET area (m^2 g^{-1})
	Fe	M	N	Fe	M	
FeMn-NC	0.569	0.479	14.98	0.36	0.37	393
FeFe-NC	1.021	-	16.44	0.67	-	405
FeNi-NC	0.556	0.432	14.09	0.34	0.30	398
FeCu-NC	0.560	0.448	12.10	0.33	0.31	373
FeZn-NC	0.530	0.453	17.06	0.29	0.30	412

Table S3. The corresponding FT-EXAFS curve fitting parameters.

Sample	Shell	CN^a	R(Å)^b	σ^2(Å^2)^c	ΔE_0(eV)^d	R factor
Zn K-edge (S_0^2 = 0.801)						
Zn foil	Zn-Zn	6*	2.66±0.01	0.0014±0.0009	7.7±0.3	0.0094
Zn	Zn-N	3.7±0.3	2.02±0.02	0.0108±0.0015	2.8±0.7	0.0129
Fe K-edge (S_0^2 = 0.783)						
Fe foil	Fe-Fe	8*	2.47±0.01	0.0038±0.0016	6.4±2.5	0.0011
	Fe-Fe	6*	2.86±0.02	0.0047±0.0032		
Fe	Fe-N	4.1±0.4	2.03±0.03	0.0089±0.0028	8.0±0.6	0.0174
Cu k-edge (S_0^2 = 0.775)						
Cu foil	Cu-Cu	12*	2.54±0.01	0.0078±0.0006	4.5±0.6	0.0093
Cu	Cu-N	4.3±0.2	1.95±0.03	0.0115±0.0019	-2.8±1.3	0.0173

^{a}*CN*, coordination number; ^{b}*R*, distance between absorber and backscatter atoms; $^c\sigma^2$, Debye-Waller factor to account for both thermal and structural disorders; $^d\Delta E_0$, inner potential correction; *R* factor indicates the goodness of the fit. S_0^2 was fixed to 0.801, 0.783 and 0.775, according to the experimental EXAFS fit of Zn foil, Fe foil and Cu foil by fixing CN as the known crystallographic value. Fitting range: 3.0 ≤ *k* (/Å) ≤ 12.5 and 1.0 ≤ *R* (Å) ≤ 3.0 (Zn foil, Fe foil and Cu foil); 1.0 ≤ *k* (/Å) ≤ 10.5 and 1.0 ≤ *R* (Å) ≤ 2.5 (Fe-N, Cu-N and Zn -N path). A reasonable range of EXAFS fitting parameters: 0.700 < S_0^2< 1.000; *CN* > 0; σ^2 > 0 Å^2; ΔE_0 < 10 eV; *R* factor < 0.02.

Table S4. Comparison table of the voltage gap (ΔE) between ORR and OER, as well as the corresponding ZAB performance of FeCu-NC and the reported SACs electrocatalysts.

Sample	ΔE (V)	Power density (mW cm^{-2})	Reference
FeFe-NC	0.68	-	This work
FeNi-NC	0.65	-	This work
FeCu-NC	0.63	231	This work
Pt/C-RuO_2	0.72	149	This work
Mn/Fe-HIB-MOF	-	195	Energ. Environ. Sci. **2019,** 12, 727-738.[14]
Co/CNFs (1000)	-	163	Adv. Mater. **2019,** 31, 1808043.[15]
Fe-N/P-C-700	-	133	J. Am. Chem. Soc. **2020,** 142, 2404-2412.[16]
SAFe-SWCNT	0.67	210	Appl. Catal. B-Environ. **2021,** 294, 120239.[17]
CNT@CoSA-Co/NCP	0.74	172	Adv. Funct. Mater. **2021,** 31, 2103360.[18]
Fe_1Co_3-NC-1100	0.70	272	ACS Catal. **2022,** 12, 1216-1227.[19]
Co-N_4/NC	0.79	101	Chem. Commun. **2021,** 57, 2049-2052.[20]
CuSA@HNCNx	0.64	212	Appl. Catal. B-Environ. **2020,** 268, 118746.[21]
FeMn-DSAC	0.71	184	Nano-Micro Lett. **2021**, 13, 60.[22]
FeCo SAs@Co/N-GC	0.64	207	ACS Nano **2021**, 15, 14683-14696.[23]
FeNi SAs/NC	0.66	-	Adv. Energy Mater. **2021,** 11, 2101242.[24]
Fe-Ni-NC-50	0.72	220	Nano Energ. **2020,** 71, 104597.[25]
Fe/Ni-N-C	0.69	-	Appl. Catal. B-Environ. **2021,** 285, 119778.[26]
0.05CoOx@PNC	0.67	157	Nano Energ. **2021,** 83, 105813.[27]
CoN_4-NiN_4-FeN_4 TSAs	0.74	132	Adv. Energy Mater. **2022**, n/a, 2203150.[28]

References

1. Ravel, B.; Newville, M., ATHENA, ARTEMIS, HEPHAESTUS: data analysis for X-ray absorption spectroscopy using IFEFFIT. *J. Synchrotron Radiat.* **2005,** *12* (4), 537-541.

2. Funke, H.; Scheinost, A. C.; Chukalina, M., Wavelet analysis of extended x-ray absorption fine structure data. *Phys. Rev. B* **2005,** *71* (9), 094110.

3. Perdew, J. P.; Burke, K.; Ernzerhof, M., Generalized Gradient Approximation Made Simple. *Phy. Rev. Lett.* **1996,** *77* (18), 3865-3868.

4. Blöchl, P. E., Projector augmented-wave method. *Phys. Rev. B* **1994,** *50* (24), 17953-17979.

5. Kresse, G.; Joubert, D., From ultrasoft pseudopotentials to the projector augmented-wave method. *Phys. Rev. B* **1999,** *59* (3), 1758-1775.

6. Kresse, G.; Furthmüller, J., Efficient iterative schemes for ab initio total-energy calculations using a plane-wave basis set. *Phys. Rev. B* **1996,** *54* (16), 11169-11186.

7. Kresse, G.; Furthmüller, J., Efficiency of ab-initio total energy calculations for metals and semiconductors using a plane-wave basis set. *Comp. Mater. Sci.* **1996,** *6* (1), 15-50.

8. Xu, H.; Cheng, D.; Cao, D.; Zeng, X. C., Erratum to. *A universal principle for a rational design of single-atom electrocatalysts (Nature Catalysis, (2018), 1, 5, (339-348), 10.1038/s41929-018-0063-z)* **2018,** *1* (8), 632.

9. Mathew, K.; Sundararaman, R.; Letchworth-Weaver, K.; Arias, T. A.; Hennig, R. G., Implicit solvation model for density-functional study of nanocrystal surfaces and reaction pathways. *J. Chem. Phys.* **2014,** *140* (8), 084106.

10. Mathew, K.; Kolluru, V. S. C.; Mula, S.; Steinmann, S. N.; Hennig, R. G., Implicit self-consistent electrolyte model in plane-wave density-functional theory. *J. Chem. Phys.* **2019,** *151* (23), 234101.

11. Wang, V.; Xu, N.; Liu, J.-C.; Tang, G.; Geng, W.-T., VASPKIT: A user-friendly interface facilitating high-throughput computing and analysis using VASP code. *Comput. Phys. Commun.* **2021,** *267*, 108033.

12. Momma, K.; Izumi, F., VESTA 3 for three-dimensional visualization of crystal, volumetric and morphology data. *J. Appl. Crystallogr.* **2011,** *44* (6), 1272-1276.

13. Nørskov, J. K.; Rossmeisl, J.; Logadottir, A.; Lindqvist, L.; Kitchin, J. R.; Bligaard, T.; Jónsson, H., Origin of the Overpotential for Oxygen Reduction at a Fuel-Cell Cathode. *J. Phys. Chem. B* **2004,** *108* (46), 17886-17892.

14. Shinde, S. S.; Lee, C. H.; Jung, J.-Y.; Wagh, N. K.; Kim, S.-H.; Kim, D.-H.; Lin, C.; Lee, S. U.; Lee, J.-H., Unveiling dual-linkage 3D hexaiminobenzene metal–organic frameworks towards long-lasting advanced reversible Zn–air batteries. *Energ. Environ. Sci.* **2019,** *12* (2), 727-738.

15. Yang, Z.; Zhao, C.; Qu, Y.; Zhou, H.; Zhou, F.; Wang, J.; Wu, Y.; Li, Y., Trifunctional Self-Supporting Cobalt-Embedded Carbon Nanotube Films for ORR, OER, and HER Triggered by Solid Diffusion from Bulk Metal. *Adv. Mater.* **2019,** *31* (12), 1808043.

16. Yuan, K.; Lützenkirchen-Hecht, D.; Li, L.; Shuai, L.; Li, Y.; Cao, R.; Qiu, M.; Zhuang, X.; Leung, M. K. H.; Chen, Y.; Scherf, U., Boosting Oxygen Reduction of Single Iron Active Sites via Geometric and Electronic Engineering: Nitrogen and Phosphorus Dual Coordination. *J. Am. Chem. Soc.* **2020,** *142* (5), 2404-2412.

17. Meng, Y.; Li, J.-C.; Zhao, S.-Y.; Shi, C.; Li, X.-Q.; Zhang, L.; Hou, P.-X.; Liu, C.; Cheng, H.-M., Fluorination-assisted preparation of self-supporting single-atom Fe-N-doped single-wall carbon nanotube film as bifunctional oxygen electrode for rechargeable Zn-Air batteries. *Appl. Catal. B-Environ.* **2021,** *294*, 120239.

18. Li, J.-C.; Meng, Y.; Zhang, L.; Li, G.; Shi, Z.; Hou, P.-X.; Liu, C.; Cheng, H.-M.; Shao, M., Dual-Phasic Carbon with Co Single Atoms and Nanoparticles as a Bifunctional Oxygen Electrocatalyst for Rechargeable Zn–Air Batteries. *Adv. Funct. Mater.* **2021,** *31* (42), 2103360.

19. He, Y.; Yang, X.; Li, Y.; Liu, L.; Guo, S.; Shu, C.; Liu, F.; Liu, Y.; Tan, Q.; Wu, G., Atomically Dispersed Fe–Co Dual Metal Sites as Bifunctional Oxygen Electrocatalysts for Rechargeable

and Flexible Zn–Air Batteries. *ACS Catal.* **2022,** *12* (2), 1216-1227.

20. Zhang, X.; Zhu, Z.; Tan, Y.; Qin, K.; Ma, F.-X.; Zhang, J., Co, Fe codoped holey carbon nanosheets as bifunctional oxygen electrocatalysts for rechargeable Zn–air batteries. *Chem. Commun.* **2021,** *57* (16), 2049-2052.

21. Wagh, N. K.; Shinde, S. S.; Lee, C. H.; Jung, J.-Y.; Kim, D.-H.; Kim, S.-H.; Lin, C.; Lee, S. U.; Lee, J.-H., Densely colonized isolated Cu-N single sites for efficient bifunctional electrocatalysts and rechargeable advanced Zn-air batteries. *Appl. Catal. B-Environ.* **2020,** *268*, 118746.

22. Chen, K.; Kim, S.; Je, M.; Choi, H.; Shi, Z.; Vladimir, N.; Kim, K. H.; Li, O. L., Ultrasonic Plasma Engineering Toward Facile Synthesis of Single-Atom M-N4/N-Doped Carbon (M = Fe, Co) as Superior Oxygen Electrocatalyst in Rechargeable Zinc–Air Batteries. *Nano-Micro Lett.* **2021,** *13* (1), 60.

23. Wagh, N. K.; Kim, D.-H.; Kim, S.-H.; Shinde, S. S.; Lee, J.-H., Heuristic Iron–Cobalt-Mediated Robust pH-Universal Oxygen Bifunctional Lusters for Reversible Aqueous and Flexible Solid-State Zn–Air Cells. *ACS Nano* **2021,** *15* (9), 14683-14696.

24. Yu, D.; Ma, Y.; Hu, F.; Lin, C.-C.; Li, L.; Chen, H.-Y.; Han, X.; Peng, S., Dual-Sites Coordination Engineering of Single Atom Catalysts for Flexible Metal–Air Batteries. *Adv. Energy Mater.* **2021,** *11* (30), 2101242.

25. Zhu, X.; Zhang, D.; Chen, C.-J.; Zhang, Q.; Liu, R.-S.; Xia, Z.; Dai, L.; Amal, R.; Lu, X., Harnessing the interplay of Fe–Ni atom pairs embedded in nitrogen-doped carbon for bifunctional oxygen electrocatalysis. *Nano Energ.* **2020,** *71*, 104597.

26. Li, H.; Wang, J.; Qi, R.; Hu, Y.; Zhang, J.; Zhao, H.; Zhang, J.; Zhao, Y., Enhanced Fe 3d delocalization and moderate spin polarization in FeNi atomic pairs for bifunctional ORR and OER electrocatalysis. *Appl. Catal. B-Environ.* **2021,** *285*, 119778.

27. Tan, Y.; Zhu, W.; Zhang, Z.; Wu, W.; Chen, R.; Mu, S.; Lv, H.; Cheng, N., Electronic tuning of confined sub-nanometer cobalt oxide clusters boosting oxygen catalysis and rechargeable Zn–air batteries.

Nano Energ. **2021,** *83*, 105813.

28. Yang, A.; Su, K.; Lei, W.; Tang, Y.; Qiu, X., Ternary Synergism of Heterogeneous $M1N_4$-C-M_2N_4-C-M_3N_4 Single-Atom Sites to Manipulate the Electrocatalytic Pathway for Zn-Air Battery and Water Splitting. *Adv. Energy Mater.* **2022,** *n/a*, 2203150.
